# Genomic medicine in hepatology: mechanisms and liver treatment strategies

**DOI:** 10.1186/s10020-025-01358-4

**Published:** 2025-09-29

**Authors:** D. S. Kozlov, S. Rodimova, P. Filatov, A. Mozherov, P. S. Timashev, M. V. Zyuzin, D. S. Kuznetsova

**Affiliations:** 1 Institute for Regenerative Medicine, Sechenov First Moscow State Medical University (Sechenov University), 8-2 Trubetskaya Str, 119991 Moscow, Russia; 2 Institute of Experimental Oncology and Biomedical Technologies, Privolzhsky Research Medical University, 10/1 Minin and Pozharsky Sq, Nizhny Novgorod, 603005 Russia; 3 School of Physics and Engineering, ITMO University, Lomonosova 9, St. Petersburg, 191002 Russia; 4 Moscow Center for Advanced Studies, Kulakova str. 20, Moscow, 123592 Russia

**Keywords:** Genomic medicine, Nucleic acid therapeutics, Hepatic fibrosis, Viral hepatitis, Nonalcoholic fatty liver disease, Targeted delivery, Gene silencing

## Abstract

Chronic liver diseases are a rapidly growing threat to global health, causing approximately 2 million deaths annually—half from complications of cirrhosis and half from viral hepatitis and hepatocellular carcinoma. Despite this, effective diagnostic and therapeutic options remain limited, prompting an urgent need for novel translational strategies. Gene-based therapeutics have emerged as a promising solution compared to conventional treatments. This review highlights the therapeutic potential of delivering nucleic acid drugs using nanoengineered materials, which take advantage of the liver’s unique anatomical and physiological characteristics. We overview the liver physiology and function, and the underlying mechanisms of gene therapy—including gene augmentation, gene silencing, and gene editing. Special attention is given to current strategies and mechanisms of gene therapy. Furthermore, we discuss the clinical translation, benefits, and limitations of gene-based approaches in treating the most widespread liver diseases and pathologies such as nonalcoholic fatty liver disease, hepatic inflammation, hepatitis, and fibrosis. By addressing the current challenges and opportunities, this review underscores the transformative potential of nanoengineered gene therapeutics in advancing liver disease treatment and shaping the future of precision hepatology.

## Background

This review explores gene-based therapeutic strategies for liver diseases, focusing on gene augmentation, silencing and editing. It discusses liver physiology, immunology and regeneration, highlighting molecular mechanisms and translational potential. Special attention is given to therapeutic approaches for nonalcoholic fatty liver disease, hepatic inflammation, hepatitis and fibrosis, emphasizing the promise of precision genomic medicine in hepatology.

## Introduction

The liver has a number of key physiological functions, including nutrient metabolism, lipid and cholesterol homeostasis, antioxidant defense, synthesis of coagulation factors, regulation of blood volume, synthesis of elements of the immune response, and endocrine control of growth signaling pathways [1, 2]. Furthermore, it also provides detoxification, performing the biotransformation of many xenobiotics and drugs [3, 4]. Given its functions and anatomic location, the liver is often exposed to various toxins such as drugs, ethanol, hepatitis B and C virus infections, and many others, and their chemical toxicity may cause acute or chronic hepatocellular injury [5]. As liver pathology develops, the following changes occur: (i) accumulation of extracellular matrix (ECM) components, (ii) impaired lipid transport with accumulation of lipid droplets in hepatocytes, (iii) inflammation, (iv) cell apoptosis and necrosis, and (v) suppression of liver regeneration. Hepatic damage also plays a significant role in the progression of any liver disease. Long-term or excessive exposure to damaging factors can lead to chronic liver injury, impaired liver function and reduced regenerative capacity. The most common liver pathologies are non-alcoholic fatty liver disease (NAFLD), its advanced stage – non-alcoholic steatohepatitis (NASH), hepatitis (viral and non-viral), fibrosis, and its decompensated form – cirrhosis, which can progress to hepatocellular carcinoma (HCC) [6]. Generally, nearly 1 million deaths are associated with liver cirrhosis and its complications, with an additional 1 million deaths due to viral hepatitis and HCC worldwide; thus, approximately 2 million people die from chronic liver diseases each year worldwide [7].

Standard methods of treating liver pathology remain insufficient. The main reason is the complexity of each liver disease, with many possible targets for therapy. In addition, since the liver is essential for the homeostasis of the entire body, inadequately selected therapy is fraught with the risk of developing systemic diseases, and even multiple organ failure and death. In this regard, liver disease therapy should be comprehensive, treating the pathology itself, stimulating liver regeneration, and restoring liver function.

**Fig. 1:**
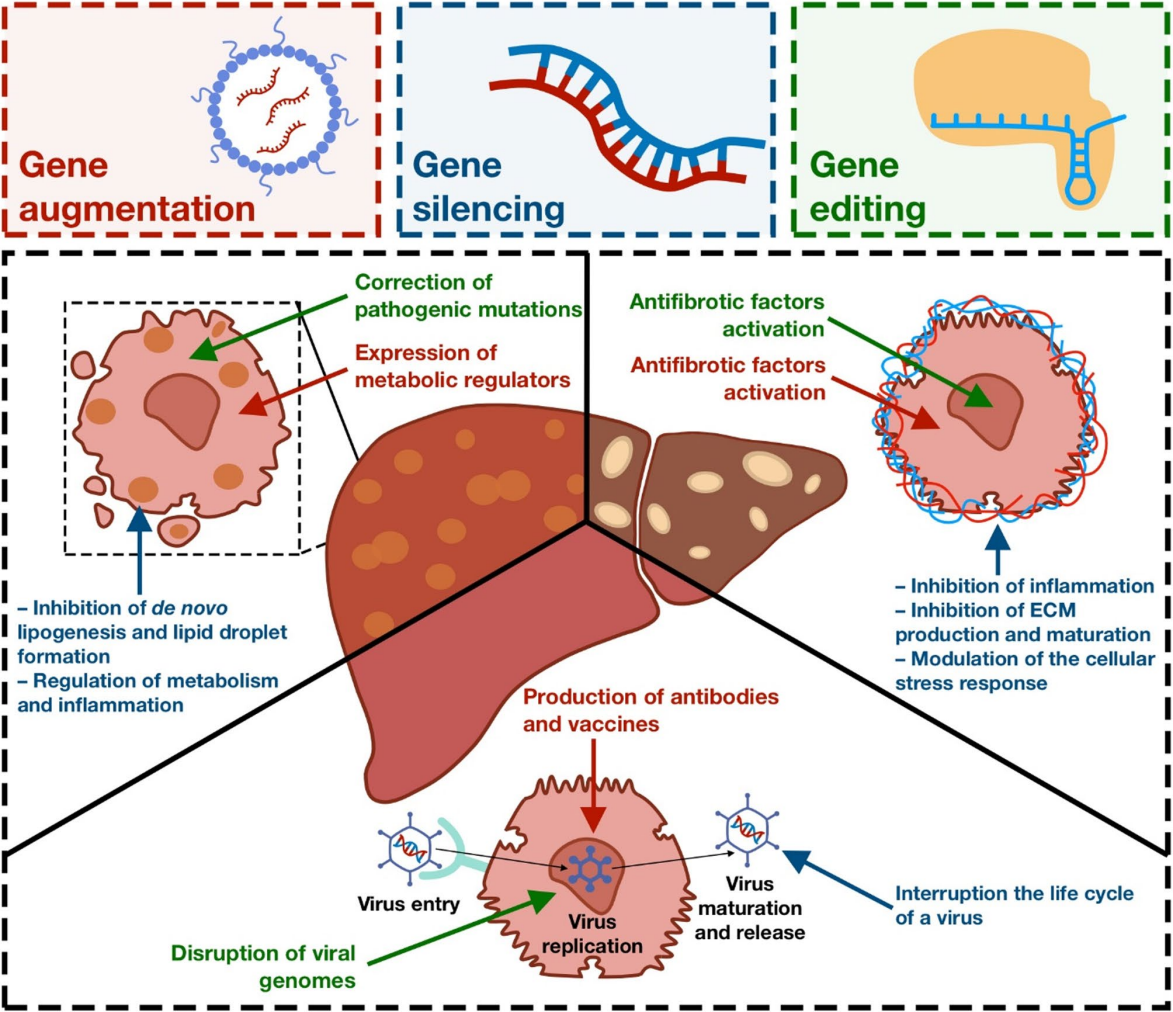
A schematic overview of genomic medicine approaches for the treatment of NAFLD, viral hepatitis and liver fibrosis

A promising alternative to the conventional liver treatment approaches is gene-based therapeutics such as delivery of DNA and RNA aimed at restoring/suppressing gene function. The latter include oligonucleotides and antisense oligonucleotide (ASO) [8], siRNA and microRNA agonists and its antagonists, which are able to regulate gene expression. Gene therapeutics can target RNA or DNA, encode missing or defective proteins, mediate DNA or RNA editing, or regulate their functional activity. However, their anionic charge and susceptibility to nucleases present in both the bloodstream and tissues make it difficult for therapeutics to enter cells efficiently and function on their own. To make therapeutics delivery safe and effective, both viral and non-viral delivery systems should be considered that could protect the therapeutics from degradation, improving delivery to target cells and minimizing side effects (Fig. 1) [9, 10]. For the treatment of liver diseases, application of viral vectors and artificial nanomaterials as carriers is expected to be the most effective due to their predisposition to primarily enter the liver, which is explained by size effects of nanosized carriers ranging from 10 nm to 1 μm [11–14]. Furthermore, to enhance the targeting abilities of nanocarriers specifically to the liver and to a specific cell type, targeting ligands are often added. The choice of a ligand specific to a particular cell type allows modulation of the functions of individual cellular components of the liver, critical for the proper functioning of many therapeutic agents, reducing the risk of complications.

Generally, viral delivery systems of gene therapeutics, which include adeno-associated virus (AAV), adenovirus (AdV), lentivirus (LV) and γ-RV based ones, are currently most used in clinics. The main advantage of viral vectors is undoubtedly the efficiency of gene delivery. However, viral delivery systems suffer from a number of limitations: viral vectors have a low packaging capacity and should be produced in accordance with GMP regulations, which demands expensive equipment and highly trained personnel. This results in large production expenses and high final cost of the product [15, 16].

Non-viral delivery systems of gene therapeutics are generally represented by organic or inorganic nanomaterials, which have a number of benefits compared to viral delivery systems. Nanoparticle (NP) carriers can be imparted with certain functions, for example, contrast properties for tracking the particles in vivo, or provide site-specific or cytosolic delivery of genes [17–20]. Although organic NPs (lipid-based NPs, polymeric NPs, cell-penetrating peptides, and others) have reached clinical trials to a higher extent, internal properties of inorganic NPs allow their use as contrast agents. For example, gold NPs provide contrast in photoacoustic or X-ray imaging, whereas magnetic NPs can be applied in magnetic resonance imaging (MRI) [21–23].

In this review, we analyze mechanisms for gene therapy of liver pathological conditions such as NAFLD, inflammation, hepatitis, and fibrosis. We provide a comprehensive guide to current strategies of liver therapy and discuss their advantages, bottlenecks, and future perspectives of gene therapeutics for liver treatment.

## Liver physiology and immunology

The liver has a central role in metabolism of nutrients (proteins, fats, carbohydrates) and removal of metabolic wastes [24]. The main liver function is to control the flow and safety of substances absorbed from the digestive system before they enter the systemic circulation [25]. The liver has a dual blood supply, which is a unique feature. The portal vein (carrying blood rich in nutrients from organs of the digestive system) provides two-thirds of the total blood flow to the liver. The hepatic artery, carrying highly oxygenated blood, supplies the rest of the blood. The hepatic veins collect blood drained by the liver and transport this blood into the inferior vena cava. The liver is the first organ receiving venous blood from the intestine (through the portal vein) [26, 27]. Due to this feature of blood circulation, and due to its detoxification function, most exogenous substances enter the liver. In the context of this review, we should highlight that the liver is responsible for the recognition and elimination of naked therapeutics, as well as viral and non-viral drug delivery vectors that enter the bloodstream. Therefore, a lot of intravenously administered substances, including gene-based therapeutics and their delivery sistems, primarily enter and accumulate in the liver [28].

The main functional unit (module) of the liver is the hepatic lobule [29]. including plates of hepatocytes, portal vessels, central vein, hepatic sinusoids running from the portal triad to the central vein, hepatic macrophages (Kupffer cells, KCs), bile canaliculi, and space of Disse – a small space between the sinusoids and hepatocytes. Portal triads consist of a small portal vein, hepatic artery, and bile duct. As blood flows through the sinusoids and space of Disse to the central vein, nutrients are processed and stored by hepatocytes. The variety of liver functions is primarily determined by the work of hepatocytes, which make up about 80% of the parenchymal mass, with non-parenchymal cells (NPCs) comprising the other 20% of the mass. Non-parenchymal cells include cholangiocytes (lining cells of the bile ducts), hepatic stellate cells (HSCs), KCs, and liver sinusoidal endothelial cells (LSECs). The remaining mass of the liver is that of the ECM. LSECs remove viruses, phages, and particles carrying exogenous ligands from the blood at an amazing rate due to their large area and a wide range of receptors [13]. These endothelial cells lack a basal lamina and are characterized by numerous fenestrae with a diameter of 150–200 nm occupying 6–8% of the endothelial surface [30]. KCs are specialized macrophages in the liver; along with LSEC, they are a key component of the reticuloendothelial system (RES), capturing and eliminating pathogens, antigens, and damage-associated molecular patterns (DAMPs), and also producing inflammatory cytokines. HSCs store vitamin A; however, if there is chronic hepatic damage, HSCs can be transformed into myofibroblasts, which synthesize components of the intercellular matrix, forming foci of fibrosis. There are conflicting data regarding the role of LSECs in the development of liver pathology. On the one hand, these cells have been shown to maintain the quiescent state of HSCs, thereby, inhibiting intrahepatic vasoconstriction and the development of fibrosis [31, 32]; on the other hand, they play a key role in the initiation and progression of chronic liver diseases [33–35]. In addition, LSECs are involved in liver regeneration after acute liver injury or partial hepatectomy due to their ability to stimulate hepatocyte proliferation. All types of cells are interconnected and play a certain role in both physiological and pathological processes affecting the entire organ.

**Fig. 2:**
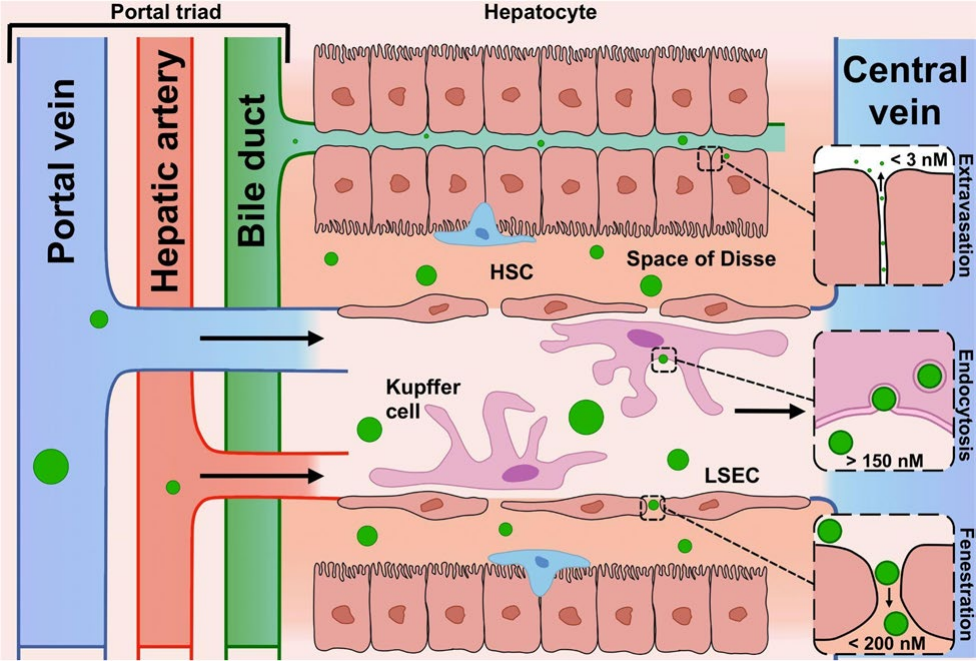
Schematic representation of mechanisms of NP biodistribution in different types of liver cells: HSC, hepatic stellate cells; LSEC, liver sinusoidal endothelial cells

Hepatobiliary elimination usually occurs through the following pathway: from the bloodstream to (1) liver sinusoid, (2) space of Disse, (3) hepatocytes, (4) bile ducts, (5) intestines, and (6) out of the body (Fig. 2) [36, 37]. Understanding the specifics of transport of substances entering the liver at the cellular level is an important aspect for the development of therapeutic drugs and their delivery systems.

The liver is a lymphoid organ with unique immunological properties, particularly its predominant innate immune system. The cellular composition of the liver is distinct from that of peripheral lymphoid organs such as the spleen, lymph nodes, and other tissues. It includes Kupffer cells (KCs), liver sinusoidal endothelial cells (LSECs), neutrophils, natural killer (NK) cells, innate lymphoid cells (ILCs), natural killer T (NKT) cells, γδ T cells and mucosal-associated invariant T (MAIT) cells, all of which function to maintain liver homeostasis [38–40]. KCs are particularly important for initiating inflammation in the liver [41]. Stimulation of KCs leads to the production of a number of cytokines IL-12, IL-6, IL-1β and IL-8, as well as TNFα, transforming growth factor beta (TGF-β), which play a key role in triggering fibrosis [42]. Moreover, TNF-α stimulates the activation of T cells, which then induce a cytotoxic response. Intrahepatic accumulation of highly activated CD8 + T cells is a part of the pathogenesis of virtually every liver disease [43]. The role of adult liver B cells remains underexplored, but B cells may aggravate fibrosis. Antibody-dependent responses of B cells contribute to liver damage, as antibody production has been shown to play a key role in alcohol-induced liver damage[44]. In addition, the liver’s immune system regulates the repair of the liver after cell injury and loss. The liver’s immune system is largely separated from the rest of the body’s immune system, but actively interacts with the intestines and peripheral organs of the immune system [45]. The immune system is an integral part of the pathogenesis of any liver disease, and therefore understanding the mechanisms of the immune response and possible ways of its regulation is necessary for effective therapeutic drug development. 

## Mechanisms of gene therapy

Currently, numerous liver-specific gene therapy approaches are under investigation or in clinical development. In recent years, a lot of efforts have been made in developing in vivo and ex vivo gene therapy solutions for the treatment of a wide range of diseases, from haploinsufficiency therapy and treatment of inherited metabolic diseases to immune deficiencies, blood disorders, solid tumors, and neurological disorders [46–57]. The success of these therapeutic approaches enables their further use for the treatment of non-hereditary diseases. Generally, gene therapy tools can be divided into three groups based on their mechanisms of action: (i) gene augmentation, (ii) gene silencing, and (iii) gene editing.

### Gene augmentation

Methods used for gene augmentation are often discussed in the context of gene replacement therapy (delivering a functional copy of a gene to replace a nonfunctional gene). However, these methods can also be used to overexpress a gene. In this regard, here we use the term gene augmentation. Gene augmentation therapy is an attractive alternative to classical methods such as growth factor therapy or enzyme replacement therapy. In some cases, a small percentage of hepatocytes carrying a genetic construct with a functional gene is required to achieve clinically significant improvements [58]. Gene augmentation therapy enables restoring a defective gene’s function, temporarily or permanently increasing the gene dose, or providing systemic secretion of monoclonal antibodies against infectious diseases or cancer [59]. This approach is effective for treating a wide spectrum of liver diseases, including hereditary disorders and viral infections.

**Fig. 3:**
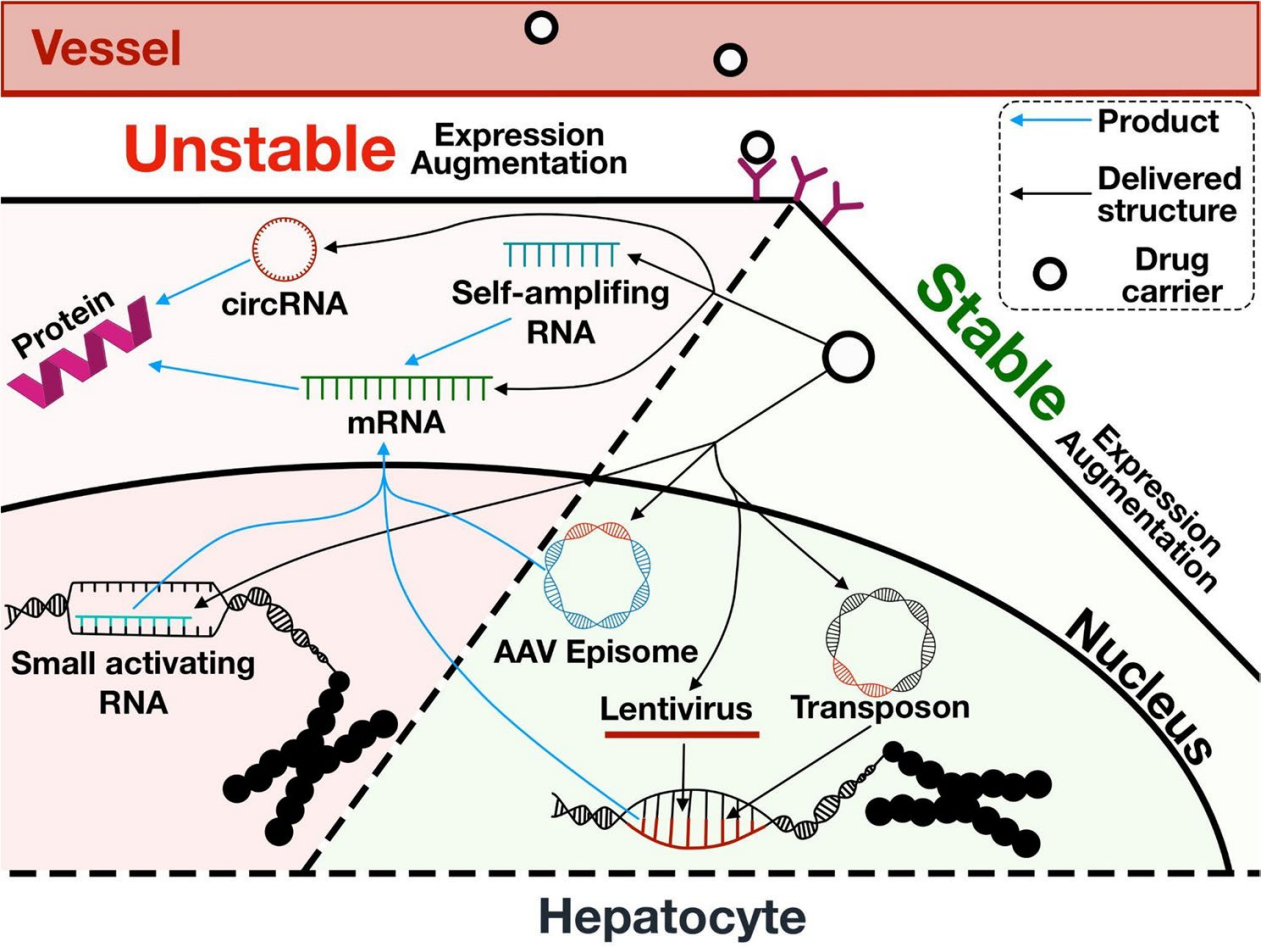
Overview of nucleic acid-based therapeutics aimed at long-term or transient gene augmentation. The modern methods allow achieving long-term constitutive expression of genes necessary for the correction of haploinsufficiency and gene replacement therapy; they include approaches such as transient hyperexpression, modulating translation efficiency, modifying the primary and secondary structure of mRNA, and increasing the stability of the introduced construct by transforming it into circRNA or self-amplifying RNA to avoid the risk of insertional mutagenesis

In the case of gene augmentation strategy, genetic material can be delivered either as DNA for long-term maintenance of function or as RNA for short-term expression and augmentation of a gene (Fig. 3). Currently, one of the most common methods of gene therapy is the delivery of an expression cassette using AAV-based vectors, which can accommodate sequences of up to 4.5–5 kb [60]. The AAV genome, upon entering the host cell nucleus, completes the second chain, subsequently undergoes ringing and concatemerization, stabilizing the vector genome for episomal persistence in postmitotic cells [61]. A single administration of an AAV vector to adult patients may be sufficient to achieve lifelong correction of diseases with a low therapeutic threshold such as hemophilia. However, gradual loss of the episomes carrying the target gene limits the therapeutic efficiency of several inherited metabolic diseases that require higher percentages of hepatocyte transduction or interventions in early childhood [62]. The reason is, hepatocytes can proliferate continuously, with individual hepatocytes surviving for about three years [63], which may substantially dilute the AAV genome and even lead to loss of transgene expression, subsequently reducing the therapeutic effect.

To overcome the disadvantages associated with transgene elimination, stabilizing sequences can be applied. Generally, stabilizing sequences can be used such as scaffold matrix attachment regions (S/MARs), which are genomic DNA sequences that link chromatin to the nuclear matrix during interphase and are involved in DNA replication and transcription [64]. DNA vectors containing the S/MAR sequence can provide long-term mitotic stability in dividing cells and avoid epigenetic silencing, allowing transgene expression to be maintained and preserving the vector stability. However, despite the initial success of this strategy in achieving robust transgene expression in the mouse and pig liver [65], S/MAR has not yet reached clinical trials.

An alternative approach is to insert the expression cassette into the host DNA using lentiviral vectors, gene editing nucleases, and transposons. Transgene stabilization can also be achieved using the transposons Sleeping Beauty (SB) and piggyBac (PB), which integrate the expression cassette into the host cell genome at transposase-specific sites [66]. However, at present, prediction of the transposon integration site remains impossible [67]. Further, despite the success of these studies, the greatest concern is the high risk of oncogenesis and disruption of gene regulatory networks due to insertional mutagenesis, which limits their application (for example, most lentivirus-based gene therapy methods involve ex vivo transduction followed by clone selection) [16].

The use of mRNA reduces the likelihood of insertional mutagenesis, and the short half-life makes therapeutic mRNAs well-suited for applications requiring transient effects, such as RNA vaccines and short-term gene augmentation. The LNP-mRNA COVID-19 vaccines, tozinameran and elasomeran, have been a landmark achievement for RNA in medicine, successfully used to vaccinate billions of people worldwide against SARS-CoV-2 to prevent COVID-19 [68]. Preclinical studies in non-human primate models, such as those for acute intermittent porphyria, further validate the safety and translatability of systemic mRNA administration [69]. Moreover, LNP-mRNA-based drugs for enzyme replacement therapy are currently undergoing clinical trials, many of which are aimed at the therapy of inherited metabolic diseases and demonstrate higher efficacy compared to standard therapies [70]. Interim results from a phase 1/2 trial of LNP-encapsulated *PCCA/PCCB* mRNAs in propionic acidaemia patients revealed reduced metabolic decompensation and good tolerability after repeated doses [71].

The intracellular stability of mRNA can be improved by modification of secondary structure and the use of base modifications including pseudouridine, N1-methylpseudouridine, and 5-methylcytosine, which in turn improves the protein production. When delivering mRNA as gene therapeutics, protein levels reach their maximum within 6–48 h after mRNA administration [72, 73]. Thus, for the treatment of inherited diseases, the duration of effect can be improved by using mRNAs that are either chemically modified [74], converted to circular RNA (circRNA) [75, 76], or contain self-amplifying sequences (saRNA) [77, 78] (recently saRNA-based COVID-19 vaccine, ARCT-154, has received approval in Japan [79]). The advantage of RNA-based therapeutics is, they do not require delivery to the nucleus to function, and the use of non-viral vectors significantly reduces the probability of a targeted immune response, allowing for repeated administration of the drug.

Another approach used for gene augmentation is the use of small activating RNAs (saRNAs), which was first described in 2006 [80]. The mechanism of action of saRNAs is not yet fully understood. Similar to siRNAs, saRNAs are synthetic, short double-stranded oligonucleotides that interact with components of the RNA-induced silencing complex (RISC) (discussed in the next section). However, unlike siRNAs, which induce transcript silencing in the cytoplasm, saRNAs are transported to the nucleus, where they target regulatory regions of specific genes. Afterwards, they promote gene expression by directly binding to DNA, promoting chromatin remodeling, and facilitating the formation of the RNA-induced transcriptional activation (RITA) complex [81].

### Gene silencing

**Fig. 4:**
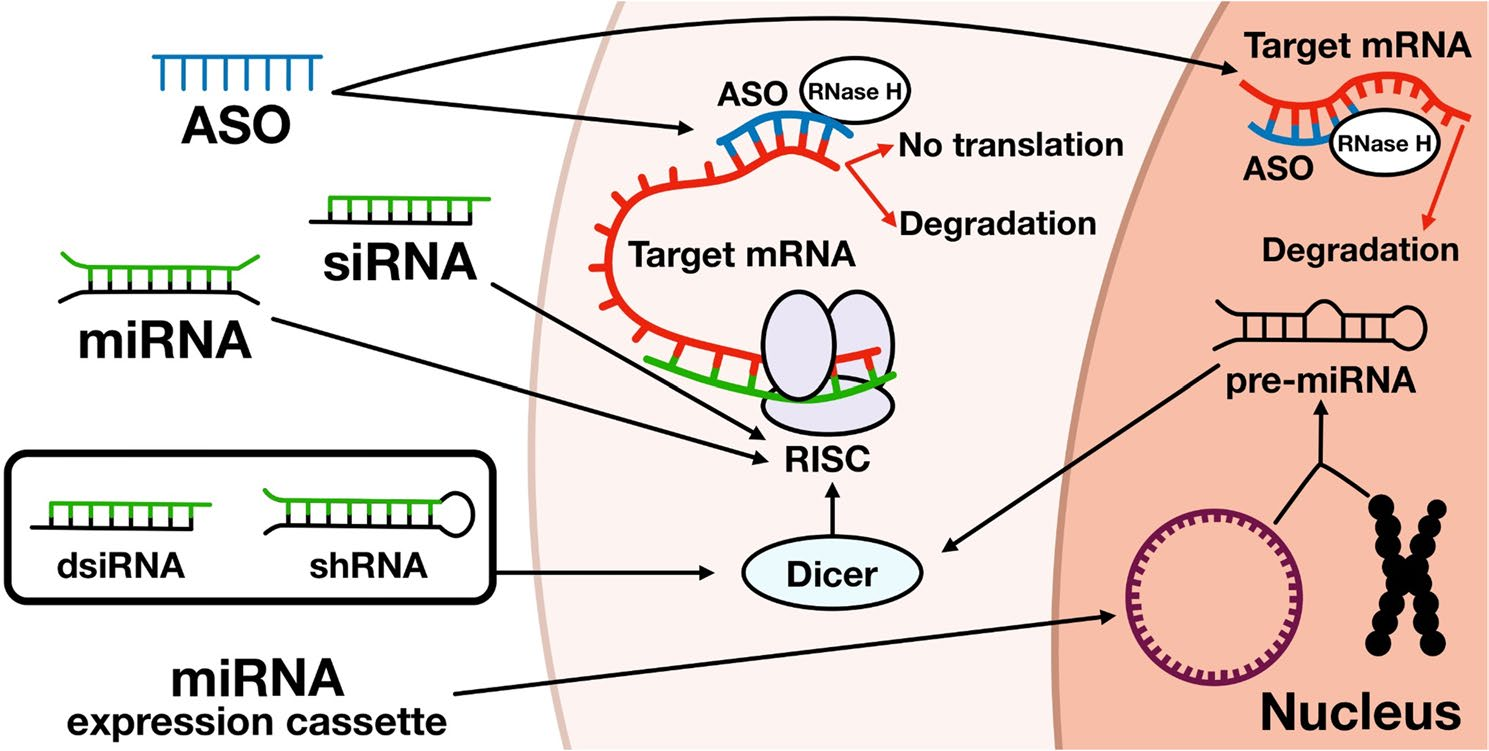
Various RNaseH- and RISC-based gene silencing approaches

Post-transcriptional gene silencing (PTGS) is a mechanism for suppressing gene expression at the level of mature mRNA, involving translational inhibition and/or mRNA degradation, but not affecting their transcriptional activity. The two most common PTGS methods include RNA interference (RNAi) (Fig. 4), which utilizes the RISC complex to cleave target mRNA, and antisense oligonucleotide (ASO)-mediated gene silencing, which directs RNase H1 to degrade target mRNA [82, 83].

ASO technology is one of the first approaches to use oligonucleotides for therapeutic purposes [84]. Since 1978, there have been several generations of modified ASOs with modulated stability and mechanism of action [85]. Currently, most ASO drugs aimed at gene silencing use RNase H1-dependent degradation of the target RNA. RNase H1 recognizes RNA–DNA heteroduplexes, cleaves the RNA strand, and releases intact DNA [86]. ASOs operating by this mechanism must have at least certain regions containing bases that are not modified at the 2′ position. For instance, there is a gapmer design, in which various 2′ and phosphate modifications can be used in the “wings”, but only 2′ OH and phosphorothioate or phosphodiester backbone modifications are allowed in the portion of the ASO (the central gap) involved in the RNase H1 catalytic mechanism [87]. Although this method is generally considered to be potent and robust with a long duration of action, Liang et al.[88] recently described the development of tolerance to ASOs. Authors found that when the recruited RNase H1 cleaves a target mRNA on the ribosome, pre-RNA levels of the same gene increase due to increased transcription, thereby reducing the gene silencing efficiency of the ASO. This tolerance process is RNase H1-dependent and target sequence-specific.

Non-cleaving ASOs can also alter translation and RNA stability by targeting critical post-transcriptional regulatory sites [89, 90]. Due to steric bulk, after pairing with mRNA, a chemically modified ASO can block transcript translation by one of the following mechanisms: (i) preventing its contact with the 40S ribosomal subunit; (ii) avoiding the assembly of the 40S/60S subunits; (iii) preventing ribosome sliding along the transcript; and (iv) preventing interaction with sequences required for transcript maturation, such as adding a cap at the 5’ end and polyadenylation at the 3’ end [91, 92].

An alternative strategy aimed at gene-specific silencing is the use of siRNAs, which use the endogenous RNA interference pathway and often demonstrate a more pronounced suppression of the target gene [93]. Similar to endogenous microRNAs, double-stranded siRNA is recruited into the RISC. Then, siRNA unwinds and separates into sense and antisense strands, and the antisense strand remains inside the complex, bound to the AGO protein. The antisense strand then binds to its complementary mRNA target, mediating cleavage and leading to degradation of the RNA molecule [93]. Exogenous siRNAs are designed to be almost completely complementary to the target mRNA and subsequently activate the endonuclease activity of AGO2, which inevitably leads to degradation of the target mRNA. In turn, endogenous microRNAs are partially complementary to the target mRNA and repress translation by destabilizing the translational complex. microRNA- RISC can also recruit enzymes that destabilize mRNA through the removal of the 5’ cap and poly-A tail [94]. Since the target binding site is short (bases 2–7 or 8 of the seed region) and there are various non-canonical binding mechanisms [95], a single microRNA can regulate more than a hundred targets. Given that the number of human microRNAs likely exceeds 2000 [96], and considering the complex and dynamic nature of microRNA–target networks [97], microRNAs are indispensable regulators of post-transcriptional gene expression. Notably, small interfering RNAs (siRNAs) and antisense oligonucleotides (ASOs) differ in structure, which leads to distinct pathways of liver cell uptake and intracellular distribution. SiRNA requires a carrier or conjugation with a targeting molecule (N-Acetylgalactosamine (GalNAc), cholesterol etc.) to be delivered into the liver cells via receptor-mediated endocytosis, whereas ASO can enter hepatocytes in its unconjugated form [98].

Breakthrough advances in the field of chemical modification of siRNA have reduced its toxicity and immunogenicity of RNA oligonucleotides, increased their stability, and improved cellular uptake by hepatocytes [99]. Currently, seven siRNA-based therapeutic drugs have been approved for clinical use, and about 20 candidates are in the late stage of clinical trials [100]. Clinical trials of siRNA currently account for about 25% of ongoing RNA therapy research. The 154 identified siRNA trials include phase 1 (25.5%), phase 2 (32.7%), and phase 3 (40.0%), with one phase 4 study. Most phase 3 trials seek additional approval for modified versions of previously approved products. However, more than half are evaluating new siRNA molecules [101].

Alternative siRNA designs that increase silencing efficiency by integrating the delivered molecules into the processing machinery are also being considered. Small interfering RNAs, which are processed by the endoribonuclease Dicer similarly to endogenous microRNAs, are an alternative to conventional 21-mer siRNAs, with an efficiency increased up to 10-fold compared to traditional constructs [102]. The loop structure of short hairpin RNAs (shRNAs) and artificially created hairpins of pri/pre-microRNA and promotes biogenesis and subsequent activity of siRNAs [103]. In turn, Dicer-substrate siRNAs (DsiRNAs) are chemically synthesized double-stranded RNAs of 25 to 27 bp in length with 2-nucleotide 3’ overhangs, which are recognized and processed into shorter 21–22 bp siRNAs by endogenous Dicer upon their introduction into mammalian cells [104]. Nucleic acids are usually used to change the expression level of microRNAs, including single-stranded anti-microRNA oligonucleotides (AMOs) (anti-miR, AntagomiR) [105], microRNA sponges [106], and others, as well as microRNA agonists: synthetic microRNAs (microRNA mimics) and recombinant expression vectors carrying sequences encoding microRNAs.

Unfortunately, the use of siRNA and microRNA as therapeutic agents for various pathological conditions is hampered by their challenging systemic delivery in vivo and potential off-target effects. Major limitations include the instability and relatively short half-life of microRNA in the blood circulation, limited cellular uptake, instability within endosomal compartments of a cell, and limited presence in the cytosol. Although several modifications of siRNA structure have been studied to improve siRNA stability, the mentioned problems have not been adequately resolved. Therefore, the development of specific microRNA carriers is of great importance [107].

### Gene editing therapy

Gene editing tools have advanced significantly in recent decades, creating new opportunities to treat many genetic disorders [108]. Using genome editing as a therapeutic strategy has the potential to reverse mutations and prevent cancer or other genetic diseases such as hepatocellular carcinoma [109, 110] and inherited liver disease [111–114]. Ex vivo genome editing, in which genes are modified in vitro prior to their re-transplantation into patients, receives the most attention, largely due to the development of CAR-T therapy [115, 116].

**Fig. 5:**
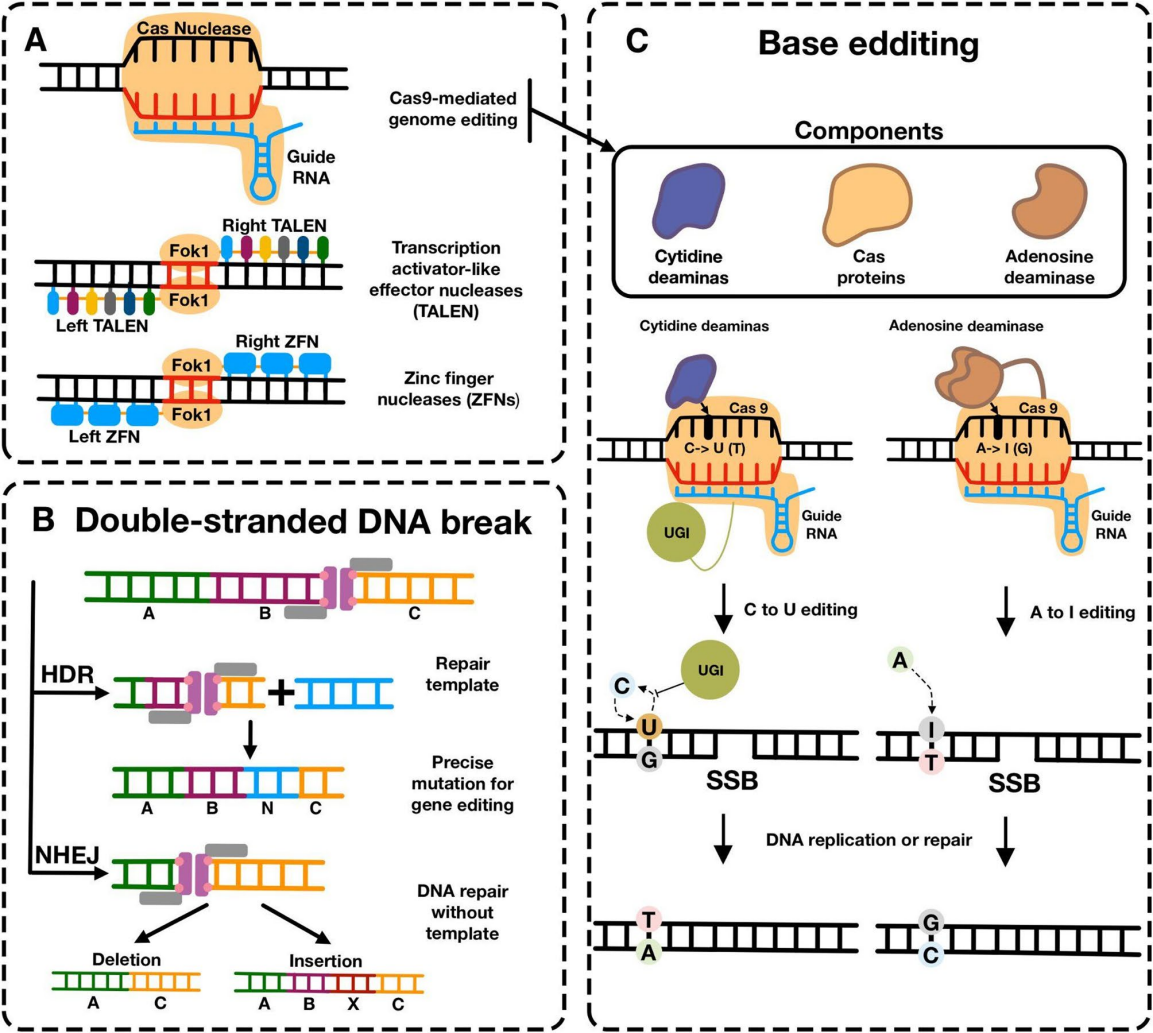
Schematic overview of gene editing strategies: **A** Site-specific nucleases induce targeted double-strand DNA breaks. **B **Base editors, derived from Cas proteins, enable precise nucleotide substitutions without inducing double-strand breaks. **C** Base editors, derived from Cas proteins, enable precise nucleotide substitutions without inducing double-strand breaks.

To date, gene editing technologies have passed three key development milestones: zinc finger nucleases (ZFN); transcription activator-like effector nucleases (TALEN); and the most widely used third-generation gene editing technology – clustered regularly interspaced short palindromic repeats (CRISPR)/CRISPR-associated protein (Cas) (Fig. 5) [117–119]. The archetypal Cas9 protein from *Streptococcus pyogenes* (SpCas9) was the first Cas nuclease repurposed for genome editing and remains the most widely used gene editor due to its intrinsically high activity and specificity [115]. Unlike ZFNs and TALENs, which use complex protein assemblies for targeted editing, CRISPR/Cas technology does not require designing a new protein architecture for each application, but merely making changes to the guide RNA (gRNA) [120].

Although each nuclease follows its own distinctive strategy, they all search for specific genomic sequences and target the region to induce double-strand DNA breaks (DSB), which will then be repaired by non-homologous end joining repair (NHEJ) or homology-directed repair (HDR). Possible gene editing mechanisms also include homology-independent targeted integration (HITI), base editing, prime editing, and nuclease-free homologous recombination [121]. Importantly, HDR is mostly active only in actively dividing cells, because it requires repair factors that are normally expressed only in the S and G2 phases of the cell cycle. Thus, the efficiency of the HDR outcome depends on the type of repair template, delivery method, cell type, local chromatin context, and other factors that may influence the choice of DNA repair pathway to preferentially enhance HDR and suppress end-joining repair [122]. Preclinical studies of HDR strategies aiming to permanently correct disease-causing mutations have demonstrated very low efficiency, unless the corrected hepatocytes received a selective advantage from the editing [123, 124] or the treatment was administered to neonatal mice. As shown in a mouse model of Fabry disease, intravenous administration of three AAV vectors carrying the α-Galactosidase (*α-Gal*) donor template and ZFN targeting the albumin locus resulted in robust supraphysiological α-Gal activity in plasma and tissues. Notably, a significant therapeutic effect was achieved with < 10% of edited hepatocytes, and the transfected hepatocytes divided more actively, which prevented the elimination of the transgene [125, 126].

Targeted delivery of gene editors remains a limiting factor for most in vivo and ex vivo gene editing applications. In particular, not only safe, specific, and efficient delivery of CRISPR components to target cells, but also the immunogenicity of CRISPR components and their vectors is challenging [127]. For most in vitro (i.e., ex vivo) applications, Cas9 and guide RNA components can be delivered as RNA, plasmid DNA, or in vitro reconstituted ribonucleoprotein (RNP) complexes. In vivo delivery of CRISPR-Cas9 is typically achieved using viral vectors. AAVs remain the preferred vectors for in vivo delivery. However, as mentioned earlier, the storage capacity of AAV is ∼4.7 kb, which makes, the delivery of SpCas9 coding sequences (4.2 kb) and its sgRNA (∼100 nt) difficult, unless ultra-compact promoters adapted from smaller Cas9 orthologs are used [128]. Finally, Cas9 mRNA combined with synthetic guide RNAs or in vitro reconstituted RNP particles can be delivered to cells in vivo, using non-viral approaches such as lipid nanoparticles (LNPs) [129].

ARCUS endonucleases have demonstrated high levels of editing at various target sites in numerous models [130–134]. In addition, ARCUS nucleases have several attractive properties for therapeutic applications, including a single-component protein containing both a site-specific DNA recognition interface and endonuclease activity. The combination of substrate recognition and catalytic motifs in a single protein encoded by ∼1100 bp allows for both viral and non-viral delivery routes and iterative improvements in both activity and specificity through protein engineering.

Since DSBs can potentially cause genotoxicity issues, nucleases are also engineered to perform single-stranded nicks (e.g., nickase Cas9 (nCas9)) or to be catalytically inactive (e.g., dead Cas9 (dCas9)). These modified Cas9s have been fused with deaminases, retrotranscriptases (RT), transcription activators, or silencing proteins to produce safer genetic or epigenetic modifications [135]. CRISPR-derived base editors (BEs) were developed as a versatile technology for generating targeted point mutations without the need to create DSBs and provide templates for homology repair, enabling editing of HDR-deficient cells. BEs are modular fusions of a nickase version of Cas9 with a nucleotide deaminase enzyme. Cytosine BEs (CBEs) mediate C-to-T conversions, while adenine BEs (ABEs) generate A-to-G conversions [136]. Recently, Liu and colleagues [137] described ‘prime editing’, which expands the CRISPR–Cas9 genome editing toolkit in critical ways. The prime editing system consists of two key components: a prime editor protein that integrates a nCas9 and a RT, and a prime editing guide RNA (pegRNA) that incorporates an additional primer binding sequence (PBS) and a reverse transcription template (RTT) at the 3′ end of gRNA [138]. Although nCas9, base editors, and prime editors can induce large deletions, their frequency is approximately 20-fold lower than that of Cas9 [139]. Prime editing has become the basis for the development of high-precision therapeutic platforms for a wide range of inherited diseases [138], increasing the accuracy of editing. Rothgangl et al. [140] employed LNPs to co-deliver PE7 editor mRNA and pegRNAs in phenylketonuria mouse model. This strategy yielded editing efficiencies of 35.9% at the *Dnmt1* locus after two LNP Doses and 8.0% at the *Pah*^*enu2*^ locus after three doses. Critically, it reduced plasma L-phenylalanine levels below the therapeutic threshold of 360 µmol l^−1^, demonstrating the potential for mRNA–LNP-mediated prime editing approaches in correcting genetic liver disorders. Also, a striking example is PRIME-Del, which induces a deletion using a pair of pegRNAs that target opposite DNA strands, programming not only the sites to be nicked but also the outcome of the repair. PRIME-Del achieves markedly higher precision than Cas9 and sgRNA pairs in programming deletions up to 10 kb, with 1–30% editing efficiency [141].

Epigenetic editors also demonstrate high therapeutic potential [142]. The CRISPR–dCas9-based epigenome editing system offers a versatile set of transcriptional or epigenetic effector domains that can be fused to dCas9 and precisely targeted to virtually any genomic locus by a gRNA complementary to the target sequence. These effector domains recruit the transcriptional machinery, altering chromatin architecture, or modifying histone residues and DNA methylation [143]. LNP-mediated delivery of mRNAs encoding engineered transcriptional repressors (ETRs) to the liver of mice led to durable (nearly one year of follow-up) epigenetic silencing of *Pcsk9 (*up to 75% of *Pcsk9* inhibition). Notably, epi-silencing remained stable even after partial hepatectomy, confirming the heritable nature of the epigenetic marks deposited by the ETR technology and the preserved proliferative capacity of epi-silenced hepatocytes [144].

#### Discussion: current limitations and research directions of genomic medicine

In the last decade, various gene therapy drugs targeting the liver have been actively investigated to correct a wide range of hereditary and acquired diseases (including metabolic diseases [62, 145–147] and tumors (especially CAR T-cell and gene therapy for HCC [148–152]). The conducted clinical and laboratory studies have revealed the pitfalls of gene therapy, associated with a wide range of issues such as immunogenicity, translation difficulties, production scaling aspects and other.

#### Immunogenicity of genomic medicine components

Immune response is a serious challenge in the development of drugs based on genomic medicine, since unmodified NAs can elicit a strong innate immune response accompanied by the secretion of inflammatory cytokines. Currently, approaches based on chemical modification of NAs, bioinformatic algorithms for identifying immunogenic sequences, and a number of other strategies are successfully applied to reduce immunogenicity [153]. Chemical modifications provide effective protection of NAs from degradation, along with reduction of immunogenicity, and increase of affinity to the target, but still possible cytotoxicity effects requires further improvement this technology [154]. In addition, despite the great potential of self-amplifying RNAs, their use is also limited due to the loss of modifications and the formation of a double-stranded structure during replication, what can provoke an immune response [155]. Unmodified circRNAs manifest fewer side effects compared with mRNA therapeutics. Even modified mRNA, designed to mitigate excessive immunogenicity, exhibits higher immunogenicity and cytotoxicity than unmodified circRNA [156].

Components of gene editing system also could be immunogenic. Specifically, neutralizing antibodies (NAbs) to both SaCas9 (*S. aureus*) and SpCas9 (*S. pyogenes*) were detected in 78% and 58% of donors, respectively. The prevalence of antigen-specific T cells against both orthologs was detected in 78% and 67% of donors, respectively [157]. A similar prevalence of preexisting immunity was also observed for enzymes Cas13d, obtained from *Ruminococcus flavefaciens* [158]. Currently, there is an active search and development of non-immunogenic nucleases that would not be recognized by adaptive immune system [159].

#### Problems and prospects of delivery systems

As mentioned earlier, the use of viral delivery systems is limited by a number of issues, including immune response to a first AAV administration, seroprevalence in patients, loss of the expression cassette in actively dividing cells, and production of NAbs. In particular, NAbs recognizing most serotypes can be found in the majority of subjects (according to various estimates, from 60 to 70% in different regions) due to the broad cross-reactivity between AAV serotypes [160]. Several strategies, including capsid engineering, plasmapheresis, enzymatic degradation of NAbs, and the use of empty capsids as baits, have been developed to address the listed problem [160, 161]. The use of non-viral delivery systems allows for multiple administration of the drug to maintain the therapeutic effect and allow usage of large expression cassettes, avoiding the risk of insertional mutagenesis.

Modularity and flexibility make LNP-mRNA-based drugs one of the most promising platforms attracting large investments, but nevertheless, a large number of issues arise related to the logistics of an unstable RNA drug, the industrial scaling, safety testing and effectiveness of the platform (to get acquainted with the proposed solutions and directions of development of mRNA-LNP drugs such as LNP chemistry, production methods and some commercial aspects, we suggest following reviews [162–164]). Besides, LNPs, especially those containing ionizable lipids, may stimulate proinflammatory cytokines and activate both antibody and T-cell responses [165–167]. And yet, some mechanisms of the immune response in model animals and humans differ, which complicates the extrapolation of results obtained in animals to humans. Androsavich [73] emphasized that in primate and mouse models, LNP-mRNA demonstrated a completely different immunogenicity profile compared to that observed in clinical trials. The probable cause of species-dependent immune responses is considered to be different levels of monocytes and/or levels of PRR expression, such as TLR3 [168] and reduced interleukin-1 receptor antagonist protein (IL-1RA) response in human cells compared to mice and primates [165]. Another problem with translational studies of LNP-mRNA is the decline in therapeutic effectiveness when transitioning from murine model to primates. Given the similar pharmacokinetic profile between different species, this may be due to subtle differences in aspects of LNP-mRNA circulation (related to opsonization, destabilization in the bloodstream) uptake, internalization and endosomal exit pathways.

Since the liver has high spatial and functional heterogeneity, it is important to consider the target characteristics, preventing off-target effects, for example, caused by ectopic expression. Thus, in the liver of mice and dogs, AAV transduction occurs predominantly in the pericentral regions, in non-human primates - in the periportal regions, and in humans, the site of AAV transduction remains unknown [169, 170]. A recent study highlighted that in a significant proportion of patients receiving gene therapy for the treatment of hemophilia, the level of AST and ALT levels was increased due to the immune response and endoplasmic reticulum stress mediated by ectopic transgene expression [171]. Despite the fact that the presence of ligands increases the targetability of the carrier, in addition to the difficulty of selecting a specific ligand, its stabilization and exposure on the surface of the carrier remains problematic.

Another aspect to consider is the formation of a protein corona, which surrounds the carrier and requires additional efforts to prevent or control its formation. А complex, dynamic, multi-component protein corona can promote opsonization and accelerated clearance of carriers, masking surface ligands, and reducing the effectiveness of targeted delivery [172–175]. Currently, various attempts are being made to prevent the formation of protein corona (“stealth nanoparticles”) that are aimed at several factors -modeling protein corona composition for active targeting (for example, Patisiran (Onpattro)) [176], stimulation the binding of deopsonizing proteins (reviewed in [177–180]) and saturation of the phagocytic activity of immune cells to prevent elimination of carriers [181–183].

Regardless of the delivery method and modality, improving endosomal release (also known as endosomal escape) would greatly enhance the efficacy of an RNA drug, but is very difficult to achieve without encountering toxicity or deterioration of other beneficial characteristics of the carrier [20]. Although the majority of LNPs (more than 95%) is endocytosed (taken up) by cells within half hour [184], less than 2% of the siRNA or mRNA escapes the endosomes to reach the cytosol [184–187].

Above, we discussed some of the challenges and directions of genomic medicine development. Despite the availability of approved platforms, delivery systems remain a bottleneck for various modalities of therapy. In Table 1, we briefly summarized the advantages and disadvantages of using viral and non-viral platforms.

**Table 1:** Comparative characteristics of viral and non-viral platforms

Feature	Viral Vectors (e.g., AAV, LV)	Non-Viral Vectors (e.g., LNPs, polymer- and metal-based NP, chemical conjugation)
Delivery Efficiency	High. Highly efficient at entering hepatocytes and delivering cargo.	Low to Moderate. Historically lower efficiency, but LNP and GalNAc-conjugates show effective liver targeting.
Duration of Gene Expression	Long-term to Permanent. AAVs provide sustained expression for years in non-dividing hepatocytes.	Transient. Expression is temporary. Requires repeat dosing for chronic effects, but suitable for acute therapies.
Risk of Genomic Integration	Low (AAV) to High (LV). Risk of insertional mutagenesis poses a long-term safety concern.	Very Low to Negligible. Do not actively integrate, offering a superior safety profile.
Immunogenicity & Hepatotoxicity	High. Can trigger immune responses causing transaminitis and, rarely, severe liver injury. Pre-existing immunity is a major barrier.	Low. Synthetic nature confers lower immunogenicity, enabling repeat administration with reduced risk of significant inflammation.
Payload/Cargo Capacity	Limited. Constrained by capsid size (e.g., AAV: ~4.7 kb).	Large and Versatile. Can carry very large genes, multiple genes, or different types of cargo (DNA, mRNA, ASOs).
Manufacturing Complexity & Cost	High. Complex, biological process. Very expensive, limiting use to rare diseases.	Low to Moderate. Simpler, synthetic process. More scalable and less expensive, enabling application to common diseases.
Suitability for Repeat Dosing	Poor. Immune response induction typically blocks re-administration.	Good. Low immunogenicity allows for repeat treatments to manage chronic liver conditions.

#### Artificial intelligence for the development of genomic medicine

The integration of artificial intelligence (AI) in the development of genomic medicine is revolutionizing the field. AI tools offer a promising approach for predicting stability and immunogenicity while simultaneously monitoring delivery system design and side effects. The AI-based approach has already been successfully applied to create modified delivery systems based on LNP [188–191], polymeric and metallic NP [192–194], AAV vectors [195–197]. Similarly, significant progress has been made using AI in determining the on- and off-target efficacy of siRNA-based drugs, which depends on factors including the spectrum of target and off-target binding sites on mRNA, competition/synergy between endogenous molecules, and others [97, 198]. This is achieved by rapidly evolving approaches of siRNA design [198, 199], and separating on- and off-target effects in throughput assays such as RNAseq [200]. However, due to interactions with endogenous RNAs and proteins, a dose optimal for a pronounced effect, which would not lead to saturation of RISC, so far can only be established in experimental settings using animal models [201, 202]. AI can also be used to predict and improve the stability and translation efficiency of RNA molecules. This depends on many factors, such as the structure of the untranslated regions, the position and strength of the IRES, the secondary structure of the RNA, the GC content, and the sequence composition [203]. AI helps to analyze the impact of all these factors and speeds up analyze huge datasets of RNA sequences and their associated stability profiles [204]. A notable example is RNAdegformer, a deep learning model that predicts mRNA degradation at nucleotide resolution. RNAdegformer outperforms previous methods in predicting the degradation properties of COVID-19 mRNA vaccines, demonstrating a stronger correlation with in vitro half-life measurements [205]. Moreover, AI-based approaches such as RhoFold + can accurately predict 3D structures of single-stranded RNAs based on their sequences, providing data on RNA stability and function [206]. Advances in AI-based tools for predicting CRISPR-Cas performance also surpassed traditional methods, offering researchers greater accuracy and speed in selecting optimal gRNAs [207]. These innovations pave the way for the creation of high-fidelity gRNAs, reducing off-target effects, and increasing overall genome editing success. In a recently published review, Abbasi et al. [208] provide detailed information on 80 available CRISPR-Cas9 related datasets that can be used to develop AI applications, giving insights into representation learning methods, trends in machine and deep learning methods, and performance values of 50 existing predictive pipelines.

#### Comorbidities as a problem for the implementation of targeted genomic therapy

Liver pathology often arises not as an independent disease but as a manifestation of systemic disorder. This also needs to be taken into account, since the targets and principles for therapy in this case change dramatically. As an example, liver disease is the leading cause of death in patients with hemophilia [171]. The success of two AAV-based liver targeting gene therapy drugs for the treatment of hemophilia A (Valoctocogene roxaparvovec) and B (Etranacogene dezaparvovec) has raised considerable interest in the hematological community, but it also has highlighted the importance of assessing liver health before and after gene therapy. Noteworthy, patients with hemophilia are characterized by a high prevalence of viral hepatitis and fatty liver disease [209]. Ragni et al. [210] emphasize that presence of steatosis or fibrosis can significantly reduce the effectiveness of gene therapy due to an increase in cell turnover and subsequent elimination of populations carrying the AAV episome or integrated cassette. Moreover, the densely organized network of ECM in the Disse space, characteristic of advanced stages of fibrosis, sterically hinders the movement of carriers, preventing the delivery of cargo [211, 212].

## Treatment of liver pathological conditions using gene therapeutics

There are many diagnostic and therapeutic drugs for pathological conditions such as non-alcoholic fatty liver disease (NAFLD), non-alcoholic steatohepatitis (NASH), hepatitis, and fibrosis (or its decompensated form – cirrhosis). However, for the successful development of liver disease therapy, it is necessary to take into account that the pathogenesis of any chronic disease is regulated by many autocrine and paracrine signals [27, 213, 214]. Therefore, affecting liver cells with a single pharmacological agent is quite difficult. The development of new strategies for the treatment of liver diseases based on the delivery of gene drugs will reduce side effects and affect individual elements of the pathogenesis of each disease.

### Nonalcoholic fatty liver disease (NAFLD)

NAFLD is one of the most common chronic liver diseases worldwide. The risk of developing NAFLD increases in case of obesity, dyslipidemia, hypertension, or insulin resistance [215]. The overall global prevalence of NAFLD has been steadily increasing, reaching 50.4% increase in the prevalence over 3 decades [216]. NAFLD can arise as a complication of metabolic diseases, most often, obesity and Type 2 Diabetes Mellitus (T2DM). Non-alcoholic fatty liver is characterized by the presence of steatosis without evidence of hepatocellular damage (ballooning) and without fibrosis. When hepatocellular ballooning and inflammation occur in addition to steatosis, the disease progresses to NASH.

In June 2023, the European Association for the Study of the Liver (EASL) Congress announced the new nomenclatures MASH (metabolic dysfunction-associated steatohepatitis) and MASLD (metabolic dysfunction-associated steatotic liver disease) to replace NASH and NAFLD, respectively [217]. In our review, we will use the terms NAFLD and NASH which broadly encompasses (> 95%) the population now recognized as MASLD and MASH [218–220], and also because most of the authors of reviewed articles use NASH and NAFLD terminology. Nevertheless, some aspects of the new nomenclature are discussed at the end of the section. Given the central role of dysregulated lipid metabolism in NAFLD/MASLD pathogenesis, our review primarily focuses on therapeutic approaches targeting this pathway, but other emerging approaches are also presented at the end of the section.

**Fig. 6:**
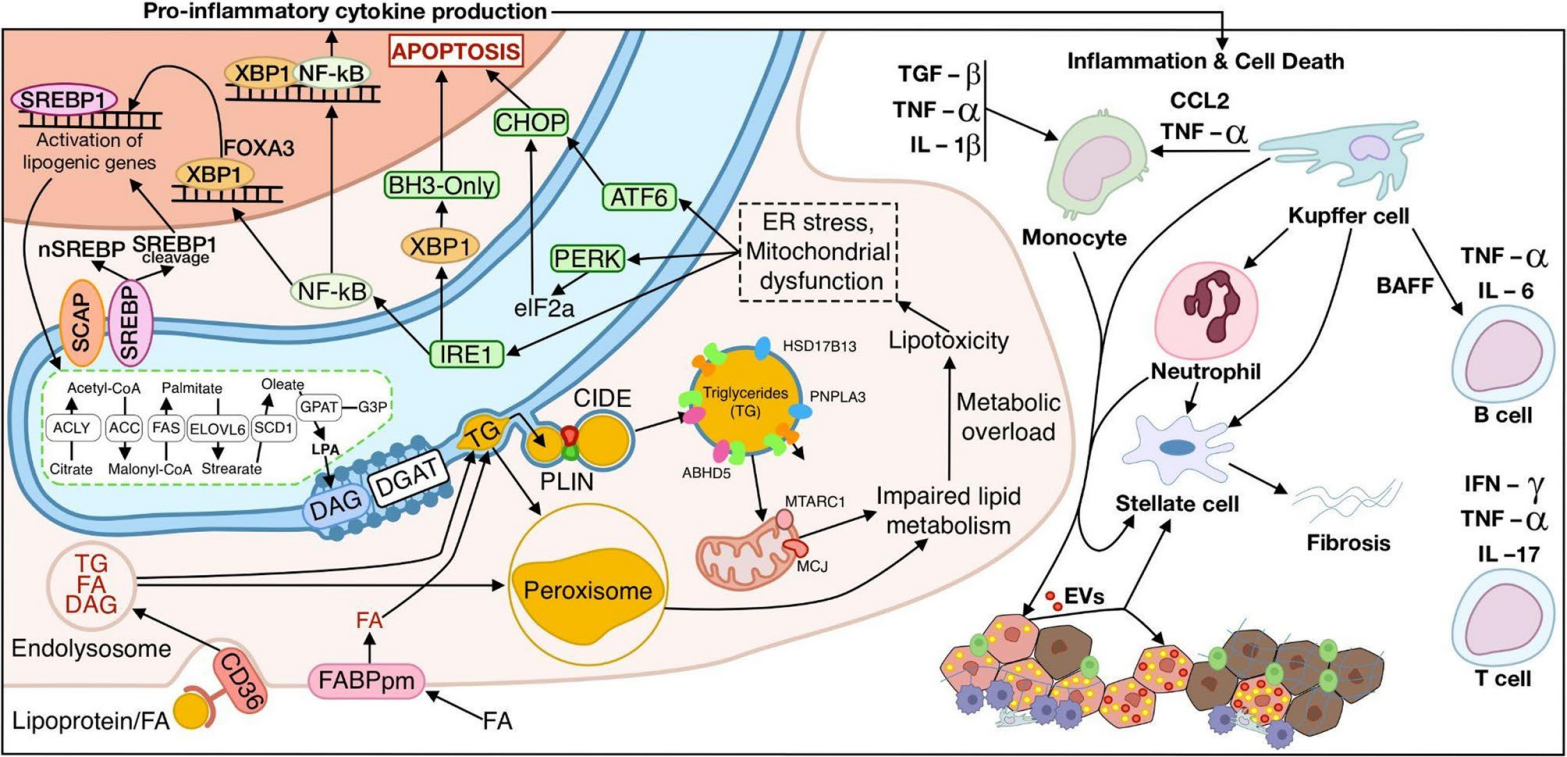
Schematic illustration of pathophysiology of NAFLD: impaired regulation of *de novo* lipogenesis; excessive formation of lipid droplets, leading to impaired lipid utilization; and mitochondrial dysfunction, which contribute to the development of ER-stress and apoptosis. Secretion of proinflammatory cytokines and DAMP, in turn, activate the immune response, aggravating the pathology

Lipotoxicity and inflammatory reactions contribute to the progression of NAFLD to NASH [221, 222]. Generally, NASH progresses in three main stages. In the first stage, lipid metabolism is enhanced, including *de novo* lipid synthesis and β-oxidation, followed by inflammation and hepatocellular apoptosis, which inevitably leads to the activation of antioxidant defense and inflammatory response [223, 224]. Chronic inflammatory response, combined with the replacement of healthy tissue with fibrous tissue, can ultimately lead to NASH [225–227]. Mechanisms of NAFLD pathogenesis complement each other, which aggravates the situation further (Fig. 6). High levels of free fatty acids in hepatocytes promote excessive mitochondrial β-oxidation, which can cause mitochondrial dysfunction, increasing oxidative stress and steatosis. In turn, the formation of reactive oxygen species (ROS) causes lipid peroxidation and can lead to oxidative damage to mitochondrial proteins, membranes, and DNA, thereby inhibiting the respiratory electron transport chain and disrupting to Ca^2+^ homeostasis. Along with this, inflammation can be developed due to excessive production of inflammatory cytokines by KCs, including TNF-α and interleukins IL-1 and IL-6; then, inflammation activates HSCs, which acquire the myofibroblast phenotype and begin to secrete collagen and ECM components [228, 229].

To date, there are no effective approaches to the treatment of NAFLD and NASH in the clinic, which makes the development of new therapeutic approaches particularly relevant. The cornerstone of NAFLD treatment is lifestyle modification and diet aimed at normalization of lipid metabolism and overall weight loss. Currently, there is only one approved pharmacotherapy for NAFLD – the THR-β agonist resmetirom. Several other classes of pharmacological agents have demonstrated efficacy in terms of histological improvement in steatosis or steatohepatitis, such as pioglitazone and glucagon-like peptide-1 (GLP-1) receptor agonists, but they have not been approved by the FDA [230]. In this regard, there is a constant search for new effective approaches to the treatment of NAFLD.

There are two main strategies on which methods proposed for the treatment of NAFLD and NASH are based. The first one is modulation of the initial mechanism of pathogenesis: decreasing lipid infiltration of hepatocytes by reducing *de novo* lipogenesis and lipid droplet formation, and stimulating lipid catabolism. The second is resolving the existing inflammation, which contributes to collagen accumulation [231–236]. It is noteworthy that genomic medicine drugs for NAFLD and NASH therapy (Table 2) largely target the same pathways as pharmacological approaches, such as small molecules, peptide mimetics and others (for detailed information, see reviews [237, 238]).

**Table 2:** Clinical trials of gene therapeutics for NAFLD treatment

Inventor	Product	Delivery system	Target	Status	Clinical trial	Results
Eli Lilly	LY3885125	GalNAc-siRNA	*SCAP*	Phase 1 (Terminated)	NCT06007651	Data not available
Ionis Pharmaceuticals	ION224	GalNAc-ASO	*DGAT2*	Phase 2	NCT04932512	at least a 2-point reduction in NAFLD Activity Score (NAS); 32% of patients treated with 120 mg achieved a ≥ 1 stage improvement in fibrosis without worsening steatohepatitis as measured by biopsy compared to 12.5% for placebo.
					NCT03334214	
Regeneron Pharmaceuticals, Alnylam Pharmaceuticals	ALN-CIDEB	GalNAc-siRNA	*CIDEB*	Phase 1	NCT06836609	Data not available
Ionis Pharmaceuticals and AstraZeneca	AZD2693/ION839	GalNAc-ASO	*PNPLA3*	Phase 2	NCT04483947	Mean knockdown of liver PNPLA3 mRNA by close to 90% from baseline; Dose-dependent increase in polyunsaturated fatty acids in circulating triglycerides; Decreased serum high-sensitivity C-reactive protein levels
					NCT04142424	
					NCT05809934	
Arrowhead Pharmaceuticals	ARO-PNPLA3/JNJ-75220795	TRIM platform		Phase 1	NCT05039710	Dose-dependent reduction in placebo-corrected Liver fat content of up to 39%
					NCT04844450	
Eli Lilly and Dicerna	LY3849891	GalNAc-siRNA		Phase 1	NCT05395481	Data not available
Amgen	AMG 609	GalNAc-siRNA		Phase 1 (Discontinued)	NCT04857606	Data not available
Regeneron Pharmaceuticals	ALN -PNP	GalNAc-siRNA(ESC+)		Phase 1	NCT05648214	Data not available
					NCT06024408	
Novo Nordisk	NN6581-4860	GalNAc-siRNA	*MTARC1*	Phase 1	NCT05599945	Data not available
Olix Pharmaceuticals Inc	OLX702A, OLX75016	GalNAc-asiRNA		Phase 1	ACTRN12624001111561	Data not available
					ACTRN12624001111561	
Novo Nordisk	NN6581, NNC0581–0001	GalNAc-siRNA		Phase 1	NCT05599945	Data not available
Regeneron Pharmaceuticals, Alnylam Pharmaceuticals	ALN-HSD	GalNAc-siRNA(ESC+)	*HSD17B13*	Phase 2	NCT04202354	At Month 6, HSD17B13 mRNA decreased by 78.3% from baseline; ALT and AST levels dropped by 11.5% and 43.6%; NAS and fibrosis stages improved over 6–12 months, reduced lobular inflammation
					NCT04565717	
					NCT05519475	
Arrowhead Pharmaceuticals	GSK-4532990/ARO-HSD	GalNAc-siRNA		Phase 1/2	NCT04202354	HSD17B13 mRNA decreased by 84% (range: 62–96%); protein levels dropped > 83%; ALT fell by 46% (range: 26–53%); 9 of 18 patients had Liver fat reductions of 4–41% on MRI-PDFF
					NCT05583344	
Ionis Pharmaceuticals and AstraZeneca	AZD7503/ION455	GalNAc-ASO		Phase 1 (Discontinued)	NCT05143905	Data not available
					NCT05560607	
					NCT05864391	
					NCT06093542	
Alnylam Pharmaceuticals	ALN-KHK	GalNAc-siRNA	*KHK*	Phase 1/2 (Terminated)	NCT05761301	Data not available
Resalis Therapeutics	RES-010	LNA-anti-miR	*miR-22-5p*	Phase 1	EUCT 2024-514871-17-00	Data not available

#### Modulation of lipogenesis

The lipid load in hepatocytes can be reduced by inhibiting the transcription factors that induce transcription of lipogenic genes and silencing the enzymes involved in lipogenesis. NAFLD progresses largely due to dysregulation of transcription factors regulating lipogenesis such as Forkhead box A3 (FOXA3), sterol regulatory element-binding proteins (SREBPs), and others.

FOXA3 is crucial in lipid and glucose homeostasis in the liver and other tissues. FOXA3 levels are elevated in the liver of obese mice and patients with NAFLD [239]. Liu et al. [240] identified FOXA3 as a key molecular mediator linking endoplasmic reticulum (ER) stress to hepatic steatosis. Suppression of *FOXA3* through the delivery of siRNA or AAV-shRNA mitigated NASH in mice with diet-induced and genetic obesity. At the same time, Gopoju et al. [241] demonstrated that AAV-mediated overexpression of FOXA3 activated lipolysis, decreased inflammation, and increased energy expenditure, thereby reducing NAFLD. These results indicate a clear relation between the FOXA3 and the mechanism of steatosis development; however, further research is needed.

**Fig. 7:**
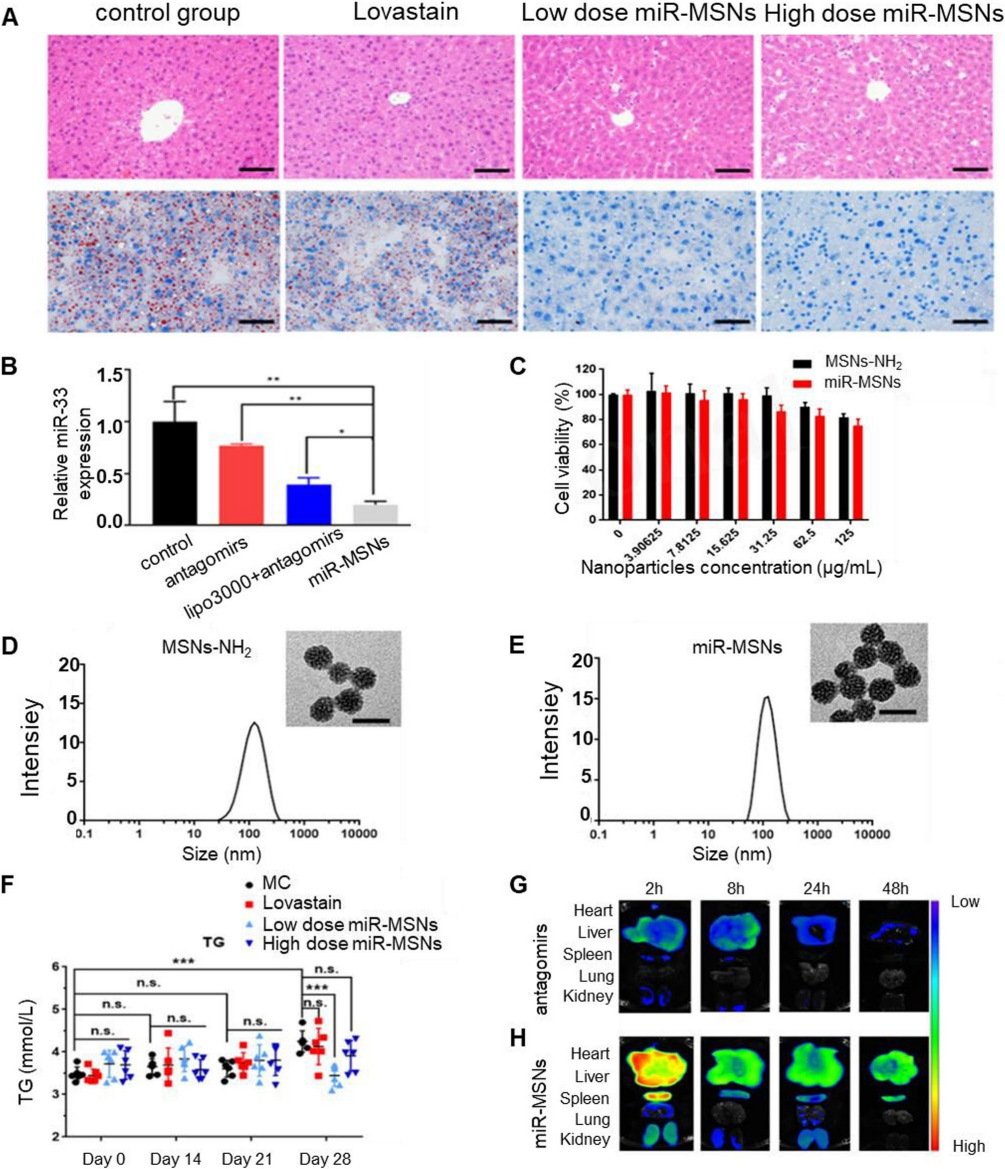
Multifaceted evaluation of miR-33 antagomir-loaded mesoporous silica nanoparticles (miR-MSNs) for the treatment of NAFLD/NASH: **A** Representative photographs of hematoxylin and eosin-stained and Oil Red O-stained liver sections (all scale bars are 50 μm). **B** Expression of miR-33 quantified by RT-PCR. **C** Cell viability assessment. **D** Hydrodynamic diameter of MSNs-NH2 and the corresponding transmission electron microscopy (TEM) image. Scale bar is 50 nm. **E** Hydrodynamic diameter of miR-MSNs and the corresponding TEM image. Scale bar is 50 nm. **F** Concentration of TG in the serum. **G**,** H** Biodistribution of nanoparticles evaluation. Ex vivo fluorescence images of major organs (the heart, liver, spleen, lung, and kidney) after the administration of antagomirs and miR-MSNs. Reproduced under terms of the CC-BY license [252]. 2020 Tao, Xu, Wang, Xu, Zhang, Chen, Lian, Zhou, Xie, Zheng and Xu, published by Frontiers Media SA.

SREBPs are transcription factors regulating the biogenesis of cholesterol, fatty acids, and triglycerides (TG) [242]. Chronic, uncontrolled activation of SREBPs increases *de novo* lipogenesis in NAFLD [101, 243]. Promising results were achieved with SREBP cleavage activating protein (SCAP) (key regulator of SREBPs maturation) targeting siRNA in rhesus monkeys. An improvement in NAFLD was confirmed by decreased hepatocyte lipid infiltration and plasma levels of TG and low-density lipoprotein cholesterol [244–246]. In August 2023, phase 1 clinical trials (NCT06007651) of *SCAP* targeting siRNA LY3885125 for patients with dyslipidemia and NAFLD started. NAT10 also promotes steatosis progression by catalyzing ac4C modification of *SERBP1c* mRNA, which leads to its stabilization and increased SERBP-1c expression [247]. The *SREBP2* and *SREBP1* genes encode miR-33a and miR-33b isoforms, respectively. Multiple studies demonstrated their role in lipid metabolism: silencing genes critical for fatty acid oxidation (Carnitine O-Octanoyltransferase (*CROT*) and acetyl-CoA acyltransferase (*HADHB*)) [248] and eliminating cholesterol due to regulation of ATP-binding cassette transporters (*ABCA1*,* ABCB11*,* ABCG5*,* ABCB4*,* ATP8B1*) and cholesterol 7 alpha-hydroxylase (*CYP7A1*) [249]. In recent in vivo studies, miR-33 deletion in hepatocytes decreased lipid synthesis and activation of mitochondrial fatty acid oxidation, reducing both lipid load and YAP/TAZ pathway activation, which also reduces the risk of carcinogenesis [250, 251]. In the work of Tao et al. [252], mesoporous silica nanoparticles (MSNs) were used as nanocarriers to deliver miR-33 antagomirs, creating microRNA-MSN nanocomposites. Authors revealed that hepatocellular uptake of miR-33 antagomirs increased approximately 5-fold when they were delivered using microRNA-MSNs. In mice fed a high-fat diet, intervention of miR-33 through microRNA-MSNs significantly reduced serum TG levels by 18.9% and reduced hepatic steatosis (Fig. 7). However, we should mention that some studies reported that SREBF1 is a direct target of miR-33-a, and thus, its inhibition would actually worsens steatosis [253, 254]. Thus, additional studies are needed to identify the role of long-term and short-term inhibition of miR-33.

FOXA3 and SREBPs activate a wide range of enzymes involved in lipid metabolism, including diacylglycerol acyltransferase 2 (DGAT2), Acetyl-CoA carboxylase (ACC), stearoyl-Coenzyme A desaturase 1 (SCD1), fatty acid synthase (FASN), and others. Dong et al. [255] showed that siRNA-mediated inhibition of *ACC1* enhanced mitochondrial respiratory function by increasing the expression of respiratory chain complexes, including NADH: ubiquinone oxidoreductase core subunit S2 (NDUFS2) and mitochondrially encoded cytochrome c oxidase II (MT-CO2), while also reducing *de novo* lipogenesis. In clinical trials, ACC-inhibiting drugs significantly decreased hepatosteatosis; however, a concomitant increase in serum triglycerides (TG) was observed, likely due to activation of sterol regulatory element-binding proteins (SREBPs) [256]. Inhibition of DGAT2 reduces *de novo* lipogenesis and helps avoid SCAP-mediated cleavage of SREBPs, also prevents and reverses triglyceride accumulation (> 85%, *p* < 0.0001), resulting in significant improvement of the fatty liver phenotype [257–259]. Two clinical trials on siRNA targeting *DGAT2* are currently underway (ION224, Ionis Pharmaceuticals, NCT04932512, NCT03334214). Phase 2 clinical trial of ION224 (NCT04932512) was completed, with positive results of reduced fibrosis in patients with NASH [260]. Inhibition of FASN, which is an enzyme synthesizing palmitate from acetyl-CoA and malonyl-CoA, also alleviates steatosis in mice with NAFLD [261–263]. Abnormal SCD1 expression and activity are associated with increased risk of various metabolic diseases, including obesity, NAFLD, and T2DM [264]. Two other clinical trials (NCT01094158, NCT02279524) in patients with NAFLD and NASH showed that Aramchol, a partial inhibitor of hepatic SCD1, significantly reduced hepatocyte lipid infiltration and improved fibrosis [265, 266]. Recent studies found that ADGRF1 acted as an upstream regulator of SCD1 and was directly correlated to fat content in obese mice and NAFLD patients. GalNAc-conjugated ASO-ADGRF1s that bind to different regions of *ADGRF1* mRNA improved glucose homeostasis, alleviating lipid abundance and liver damage in mouse model [267]. Also, siRNA targeting *SCD1* reduced the lipid infiltration of hepatocytes and stimulated lipophagy [268, 269]. Liu et al. [269] demonstrated that restoring miR-192-5p levels could also decrease the SCD1 expression and lipid accumulation. However, at the same time, SCD1 prevents lipotoxicity by desaturating fatty acids [270]. Further, miR-192-5p is related to liver development and cancerogenesis; it is a marker of hepatocyte death in liver injury [271]; the significance of this microRNA was also confirmed in a recent study Ma et al. [272], in which miR-192-5p regulated *de novo* lipogenesis via modulation of FASN activity mediated by targeting *Yin Yang*1 (Yy1), demonstrating that overexpression of miR-192-5p significantly reduces TG levels both in vitro and in vivo.

#### Prevention of lipid accumulation

Another possible target for therapy is the accumulation of lipid droplets in hepatocytes, which leads to lipid oxidation, lipotoxicity, decreased lipid utilization, and activation of endoplasmic reticulum (ER) stress response pathways [273].

Overexpression of perilipins (PLINs) causes hepatocyte ballooning when PLINs interact with fat-specific protein 27 (Fsp27) (also known as cell death-inducing DFFA-like effector C (CIDEC)), which mediates the formation and stabilization of lipid droplets [274–276]. siRNA and shRNA-mediated knockdown of *Fsp27* reduces accumulation of TG and lipid droplets formation in hepatocytes [277–280]. Another member of the CIDE protein family, CIDEB, has also been implicated in the progression of NASH by promoting lipid droplet formation and participating in SREBP maturation [281]. Currently, preclinical studies — including those conducted by Regeneron Pharmaceuticals and Shanghai Argo Biopharma — are actively exploring siRNA-based approaches targeting *CIDEB*, with promising results reported to date [282–284]. Recently, Wang et al. [285] revealed that GalNAc-siRNA targeting *Plin2* decreased hepatic TG level and the accumulation of lipid droplets by 60% in mice. Genome-wide association studies (GWAS) conducted in multiethnic cohorts have identified a number of genes associated with lipid metabolism and the development of steatosis. Mutations in these genes increase the risk of developing NASH. The rs738409 C > G variant (frequency ranges from 17 to 49%) in the patatin-like phospholipase domain containing 3 (*PNPLA3*) gene, encoding PNPLA3 I148M, is the strongest genetic variant predisposing to the development of steatosis and its progression to NASH and HCC [286–288]. The mutant form of PNPLA3 (I148M) was shown to prevent the activation of lipolysis [243, 289]. Currently, a large number of ASO and siRNA therapeutics targeting *PNPLA3* have entered clinical trials (GalNAc-ASO AZD2693/ION839 (Phase II) NCT05809934 [290]; ARO-PNPLA3/JNJ-75220795 (Phase I) NCT04844450 [291]; LY3849891 (Phase I) NCT05395481; AMG 609 (Phase I) NCT04857606; ALN -PNP (Phase I) NCT05648214.

Another promising approach is silencing of genes of mitochondrial amidoxime-reducing component 1 (MTARC1) [292] and 17β-hydroxysteroid dehydrogenases (HSD17B13) [276]. MTARC1 overexpression is associated with mitochondrial dysfunction and increased ROS production [293]. Generally, GalNAc-siRNA NN6581 targeting *MTARC1* has entered phase I clinical trials (NCT05599945), as well as GalNAc-modified asymmetric siRNA (GalNAc-asiRNA) OLX75016 (the candidate of OLX702A) (ACTRN12624001111561, ACTRN12624001111561). In preclinical studies, OLX702A demonstrated significantly decreased liver inflammation, fat content, and fibrosis in a non-human primate NASH model [294]. Molecular function of HSD17B13 remains unclear since its discovery, but HSD17B13 is suggested to be involved in the formation and stabilization of lipid droplets [275]. *HSD17B13* siRNA inhibitors are also undergoing clinical trials (ALN-HSD NCT04202354, NCT04565717, NCT05519475, GSK-4532990/ARO-HSD (Phase II) NCT04202354, NCT05583344, AZD7503/ION455 (Phase I) NCT05143905, NCT05560607, and NCT05864391) with highly prommising results [276, 295].

Fatty acid catabolism can be activated through the silencing of Methylation-Controlled J protein (MCJ, also known as DNAJC15), negative regulator of mitochondrial respiration [296–298]. Increased MCJ expression has been observed in NAFLD patients, whereas MCJ deficiency in mice is associated with reduced liver steatosis and fibrosis [299]. Decrease in MCJ enhances the activity of the complex I of the electron transport chain, as well as mitochondrial respiration [297]. *MCJ*-targeted siRNA delivered with LNPs reduced lipid accumulation, fibrosis, and hepatocyte damage in NAFLD models [299, 300]. A recent study has shown that suppression *MTFP1* (Mitochondrial Fission Process 1) by siRNA leads to the upregulation of oxidative phosphorylation activity and mitochondrial respiration and also inhibits mitochondrial permeability transition pore opening in hepatocytes, conferring protection against high fat diet-induced steatosis in vivo and ex vivo [301]. Restoration of the level of hepatocyte nuclear factor 4 alpha (HNF4a) may be another approach to the treatment of NAFLD [302]. Huang et al. [303] applied small activating RNA technology to activate *HNF4a *in vitro and in vivo. As a result, genes involved in beta-oxidation of fatty acids and lipid transport were significantly regulated, decreasing cholesterol, TG content, and the level of pro-inflammatory IL1b in the liver.

The levels of lipid droplet-associated protein serine/threonine protein kinase 25 (Stk25) are associated with an increased activity of cytoplasmic peroxisomes and the development of oxidative and ER stress [304] and correlate with the severity of steatosis in humans and model organisms [305, 306]. Several research groups showed the therapeutic applicability of ASO targeting *STK25* [304, 307]. For instance, the STK25 ASO is currently under preclinical study by Sprint Bioscience. Chemical modified ASOs targeting mammalian sterile 20-like 3 (*MST3*, also known as *Stk24*) have also shown the capacity to ameliorate diet-induced NAFLD, including the reduced oxidative stress and ER stress biomarkers (4-hydroxynonenal, 8-oxoguanine, KDEL, and CHOP) in mouse livers [308]. Chen et al. [309] also demonstrated the role of miR-149-mimics in alleviating inflammation and cell apoptosis in a mouse model of NAFLD by inhibiting the ER stress signaling pathway through regulation of specific protein activating transcription factor 6 (ATF6).

An obvious and promising way for the treatment of NAFLD is modulating the expression of various growth factors. Injection of *HGF* (hepatocyte growth factor) mRNA encapsulated in LNPs significantly reduces hepatocyte damage and ballooning in the mouse model of NAFLD, restoring the liver function (ALT returned to normal baseline levels) [310]. Recently, the hormone fibroblast growth factor 21 (FGF21) has attracted attention as a possible therapeutic agent for the treatment of metabolic disorders, including NAFLD [311]. An increased expression of FGF21 enhances lipolysis and lipid catabolism in hepatocytes [312, 313]. However, due to the short half-life and biophysical disadvantages of the native FGF21 peptide [314], researchers have focused on using AAV vectors to deliver *FGF21* for long-term and sustainable protein production in vivo. Jimenez et al. demonstrated that administration of *FGF21* using the AAV vector to mice with steatosis has improved the energy metabolism, reduced adipocyte size and inflammation for over a year, and, as a consequence, decreased the body weight [315]. Bartesaghi et al. [316] and Huang et al.[317] subcutaneously injected LNPs loaded with *FGF21* mRNA into mice, which significantly decreased the hepatocyte lipid infiltration and TG levels, led to weight loss, and increased energy consumption.

#### Bile acids metabolism

Recently the role of gut–liver axis in progression of NAFLD/NASH garnered significant attention [318–323]. Bile acids (BAs) metabolism plays an important role in this axis. High levels of bile acids are often observed in patients with NAFLD. The meta-analysis [324] of 19 studies involving 154,807 people found that levels of four specific bile acids were significantly elevated in patients with NASH. The reason is the close relationship between lipid and BAs metabolism. Disrupted balance of BAs synthesis (via cholesterol 7α-hydroxylase (CYP7A1)), transport (ABCG5/8/B11 and SLC10A1) and signaling pathways (primarily regulated by the farnesoid X receptor (FXR) and the liver X receptor (LXR)) in turn affects the lipogenesis and the gluconeogenesis [325, 326], and provokes inflammation [327, 328].

Managing bile acid metabolism may be a promising strategy for primary or adjunctive therapy in NAFLD and NASH [329]. Examples of drugs aimed at controlling the metabolism of fatty acids and BAs are FXR, LXR agonists and FGF19 mimetics. FGF19 is a gut hormone that inhibits BA synthesis from cholesterol via cytochrome CYP7A1 and reduces insulin-induced hepatic lipogenesis. FGF-19 has structural similarities to FGF-21 and activates an overlapping set of receptors [330]. It was shown that FGF19 associated with the NAFLD pathogenesis, as it affects lipid and glucose metabolism and is involved in inflammation [331]. One of FGF-19-mimicking therapeutics (aldafermin) was recently tested in clinical trials for NASH therapy [332]. In a cohort of 200 patients with NASH with F2 or F3 fibrosis, aldafermin administration led to fibrosis improvement by one stage in 31% of patients compared with 19% in control group. Co-administration of FGF-19-type agents with statins can mitigate the effect of elevating low-density lipoprotein cholesterol levels [333]. Delivery of the engineered nontumorigenic FGF19 transgene using AAV vector [334] and mRNA-LNPs [335] are also resulted in a rapid reduction in ALT and AST levels and a strong improvement in histopathological abnormalities, including hepatic steatosis, inflammation and ballooning degeneration.

#### Insulin resistance

The relationship between insulin resistance (IR) and the pathogenesis of NAFLD and NASH has been widely reported [336–339]. Crucially, IR is a well-established driver of liver steatosis and fibrosis progression, primarily through disturbances in lipid metabolism: promotes white adipose tissue lipolysis that enhance portal delivery of lipid metabolites (glycerol and fatty acids) to the liver [340], stimulates hepatic *de novo* lipogenesis, and alters mitochondrial fatty acid β-oxidation (FAO) [341]. Initially, hyperinsulinaemia and hyperglycaemia in NAFLD directly exacerbate lipid accumulation. More specifically, SREBP1 and ChREBP are activated by disruptions in canonical insulin signaling, activation of non-canonical pathways, and hyperglycemia, leading to activation of *de novo* lipogenesis -related genes in the liver [341–343]. Furthermore mitochondrial FAO increases to compensate for heightened lipogenesis. However, this adaptation eventually decompensates, leading to mitochondrial damage, oxidative stress, and impairment of insulin signaling thereby reinforcing a vicious cycle [344].

The approaches for reducing the manifestation of IR based on genomic medicine mainly aimed at maintaining glucose homeostasis [345], restoring transcription factors expression (e.g. HGF [346]) and reducing lipotoxicity (e.g. by targeting *PLIN2* [285]). Also, siRNA mediated knockdown of the cysteine-rich 61 (*Cyr61*) [347] or *TOX4* [348] which aimed at treatment of hyperglycemia, impaired glucose tolerance and IR, simultaneously improved lipotoxicity and inflammation in liver. A study by Ding et al. [349] used clinically approved LNP to encapsulate siRNA targeting hepatic ubiquitin-specific peptidase 20 (*USP20*). Authors showed that *USP20* inhibition reduced fat composition by ∼10% in serum and liver, improved glucose clearance and insulin sensitivity, and increased energy expenditure through elevated UCP1 in brown and white adipose tissue (BAT/WAT) in mouse model. This approach holds promise for metabolic syndrome treatment. Preclinical studies of ALN-KHK, a GalNAc-conjugated siRNA targeting hepatic ketohexokinase (*KHK*), demonstrated decreased hepatic lipogenesis and increased insulin sensitivity, thereby improving glycemic control in obese individuals with type 2 diabetes [350]. However, the phase 1/2 study of ALN-KHK (NCT05761301) was recently terminated.

Notably, many microRNA therapeutics in development for NAFLD directly or indirectly target the same key proteins (GLP-1R, THR-β, SGLT1/2, PPAR, FGF21 and FXR) as small-molecule agonists undergoing trials. Targeting these proteins has shown promising results in alleviating NAFLD and improving insulin sensitivity. For example, targeting miR-132, which regulates *PTEN*, *FOXO3*, and *SIRT1* more effectively than targeting its individual targets [351]. Based on these results, Regulus Therapeutics Inc. developed anti-miR-132, demonstrating improvements in hyperglycemia, hyperlipidemia, metabolic gene expression and ALT, AST levels in NASH models [352], although study updates remain unavailable. Inhibition of miR-22, which targets *FGF21*, *PPARα*, *SIRT1*, and *PGC-1α* resulted in decreased triglyceride levels and alleviation of steatosis and IR [353, 354]. In this regard, Resalis Therapeutics developed an LNA-based anti-miR-22-5p (RES-010), which restores the regulation of lipid biosynthesis and increases energy expenditure, resulting in effective reduction of steatosis, fibrosis and inflammation [353, 355]. RES-010 has entered phase 1 clinical trials (EUCT 2024-514871-17-00), and its GalNAc-conjugated derivative RES-020 is now in development. Another example is the miR-10b-5p mimic RSVI-301 (RosVivo Therapeutics), which increases receptor tyrosine kinase (RTK) expression by downregulating Krüppel factor 11 (*Klf11*), improved glucose homeostasis, thereby reducing IR in mice [356, 357].

#### Inflammation

Macrophage polarization and toll-like receptor (TLR) signaling are components of the inflammatory response contributing to progression of NASH; related approaches are currently being investigated for the treatment of inflammation in NAFLD.

**Fig. 8:**
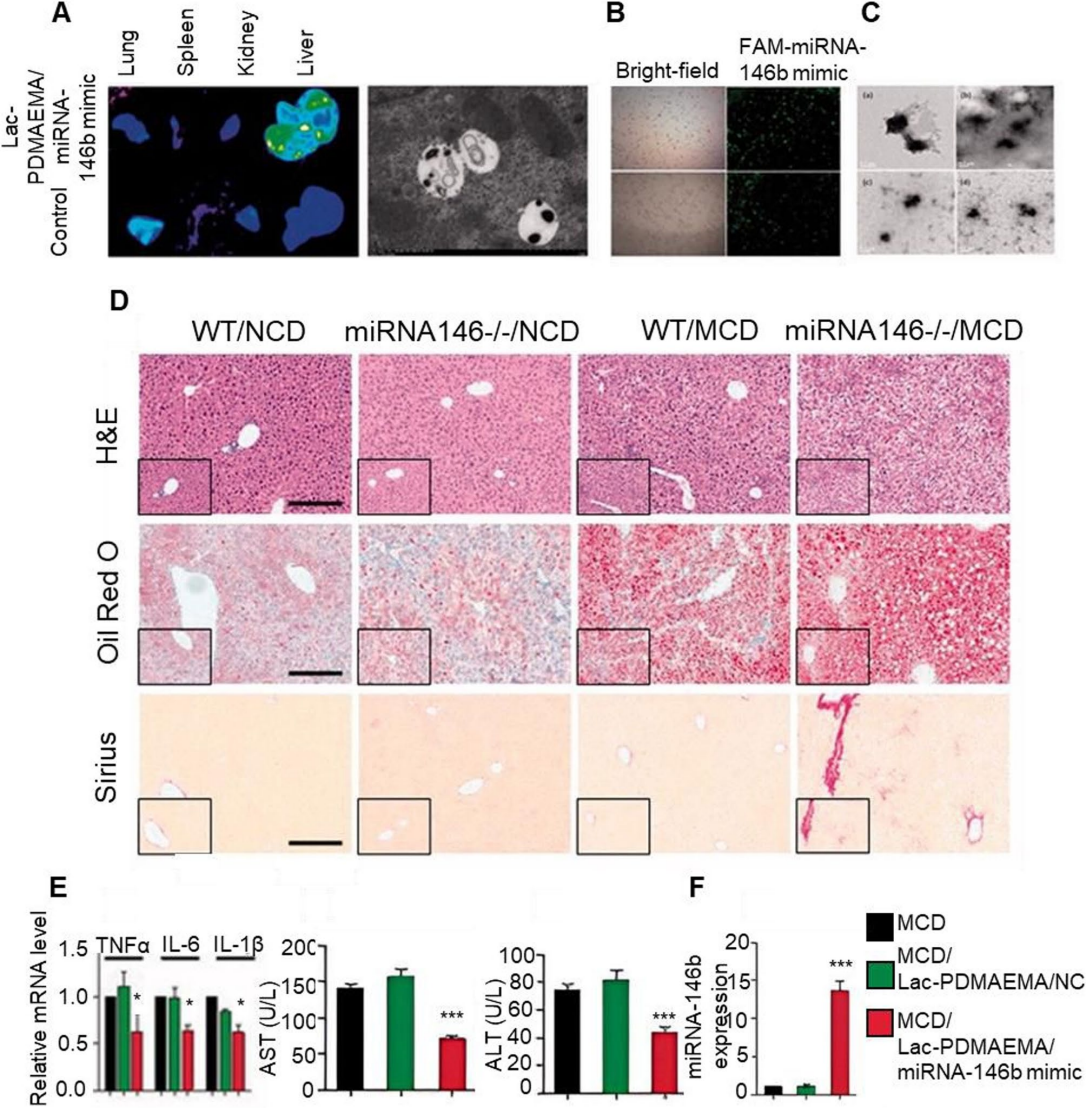
Anti-inflammatory effects of miR-146b mimic delivered via lactosylated nanoparticles: **A** Tissue distribution of Lac- PDMAEMA/Cy3-miR-146b mimic in mice. Morphology of Lac-PDMAEMA/Cy3-miR-146b mimic determined by TEM in Liver. Scale bar represents 100nm. **B** Uptake effect of Lac-PDMAEMA/miR-146b mimic complex in HepG2 and AML12 cell lines **C** TEM images of the Lac-PDMAEMA/miRNA complexes. **D** Representative H&E-stained (left) and Oil Red O-stained (right) liver sections from WT and miR-146b−/− mice that were fed an methionine–choline-deficient diet (MCD) for 3 weeks. Scale bar is 100 μm. **E** qPCR analysis for TNF-α, IL-6, and IL-1β expression in liver samples. Gene expression was normalized to β-actin. Serum levels of ALT and AST. Data are shown as the mean value ± SD; *p < 0.05, **p < 0.01, ***p < 0.001 versus the MCD group. **F** Efficacy of transfection of miR-146b mimic determined by qPCR. Reproduced with permission [364]. 2018, published by Taylor & Francis.

Several microRNAs have been identified as regulators of TLR signaling, including miR-181-3p, miR-146b, miR-140, miR-204-3p, and miR-16 [358–363]/He et al. [364] applied lactosylated LNP containing mimic miR-146b as carriers for the delivery to the liver. This decreased the production of *TNF-α* and *IL-6* mRNA levels by downregulation key TLR4 signaling factors MyD88 and Interleukin 1 Receptor Associated Kinase 1 (IRAK1) (Fig. 8), which was also confirmed in the work of Curtale et al. [365]. Phase 2 clinical trial of ASO targeting *TLR9* (AVO101) causes elevations in adiponectin, followed by NASH resolution in an obese primate model [366]. Ouyang et al.[367] employed *SCAP*-targeting siRNA and demonstrated activation of the proinflammatory NF-κB signaling pathway. *SCAP* silencing decreased the expression of pro-IL-1β, TNF-α, monocyte chemotactic protein 1 (MCP-1), and mature IL-1β in macrophages. Calvente et al. [368] delivered the LNP miR-223-3p to prevent activation of the NLR family pyrin domain-containing 3 (NLRP3) inflammasome and significantly reduced the infiltration of monocytes, neutrophils, and early activated macrophages in NASH. He et al. [369] used mannose-modified trimethyl chitosan-cysteine (MTC) conjugate NPs for the delivery of siRNA to inhibit production of inflammatory cytokine *TNF-α* by macrophage in vivo, preventing the activation of HSCs. The work demonstrates a significant decrease in the TNF-α production, a 43% decrease in the alpha smooth muscle actin (*a-SMA*) mRNA expression, and a 81% decrease in *COL1A1* mRNA expression in the liver.

Tripathi et al. [370] used LNPs to deliver siRNA that suppresses the pro-inflammatory expression of runt-related transcription factor 1 (*RUNX1*), decreasing the infiltrated T cells and myeloid cells and reducing the inflammatory cytokines in the liver of *RUNX1* siRNA treated mice, which ultimately reduced the NASH progression.

Reprogramming macrophages from the anti-inflammatory M1 phenotype to the pro-regenerative M2 phenotype is considered a promising approach for the treatment of a wide range of diseases [371]. Zhou et al. [273] used a mannose-modified LNPs loaded with HMGB1-siRNA (mLNP-siHMGB1) to target liver macrophages, restoring the liver function by silencing high-mobility group Box 1 (*HMGB1*), which reduced inflammation and promoted the transformation of the proinflammatory M1-type macrophages into the anti-inflammatory M2-type. Similarly, Zhu et al.[372] employed folic acid-modified D-α-tocopherol (FT@XBP1) NPs to deliver X-Box binding protein 1 (*XBP1*) targeting siRNA, which promoted macrophage polarization toward the M2 phenotype and enhanced the release of exosomes that inhibited the activation of HSCs.

Targeting liver macrophages with different variants of CRISPR aimed at inactivating pro-inflammatory factors also represents an interesting approach. In a recent study Wu at al. [373] developed a novel delivery system by encapsulating CRISPR/Cas9 RNPs within mannosylated neutrophil membranes vesicles (Cas9/gNLRP3@M-N) to enhance targeting hepatic macrophages. Using this system, effective disruption of the *NLRP3* gene in hepatic macrophages attenuated inflammation in acute liver failure, and improved outcomes in chronic NASH model. Macrophage-specific knockout of *NLRP3* not only reduced inflammation (confirmed by reduced levels of IL-1β, TNF-α, and IL-18) but also downregulated the expression of genes involved in fatty acid synthesis and uptake (SREBP1, FASN, PPARα, and CPT1A). Therapy significantly improved microvesicular and macrovesicular fat deposition, tissue morphology and normalized ALT and AST levels.

#### Discussion: current limitations and research directions of NAFLD treatment

Recently, a Delphi consensus led by multiple professional societies announced updated nomenclature and the refinement of its definition to allow appropriate classification in patient identification [374]. Central to this change is the shift from “fatty liver disease” to “steatotic liver disease”, which includes all conditions characterized by abnormal hepatic lipid accumulation and encompasses MASLD, alcohol-associated liver disease (ALD), metabolic dysfunction- and alcohol-associated liver disease (MetALD) and other rare causes of liver steatosis [375–377]. Patients presenting with cardiometabolic risk factors such as obesity, insulin resistance, diabetes, dyslipidemia and alcohol intake below 20 g/day for women or 30 g/day for men are now diagnosed with MASLD [217]. This new nomenclature embraces a more comprehensive and nuanced approach to classifying liver diseases and opens up new avenues for various medical fields, including primary care, internal medicine, hepatology, drug development and other fields [332, 378, 379].

Many approaches aimed at steatotic liver disease therapy have demonstrated synergy with drugs for the treatment of obesity, diabetes and cardiovascular diseases, and vice versa (for example, commonly used cholesterol-lowering drugs statins demonstrate benefits in ameliorating MASH [380, 381]). On the contrary, statins can also activate SREBP pathway and subsequently upregulate HMGCR expression, thereby promoting *de novo* lipogenesis [382, 383]. Previously described siRNA-Usp20 completely blunted statin-induced HMGCR increase and showed decline in lipid accumulation [349]. Furthermore, preclinical studies previously described *MTARC1* targeting siRNA OLX702A and anti-miR-22-5p (RES-010) combining with the anti-obesity drug semaglutide (GLP-1R agonist) demonstrated greater reduction in weight loss compared to semaglutide alone.

A combined approach that targets multiple pathways can increase the effectiveness of therapy, reduce therapeutic doses, and mitigate drug side effects. Although monotherapy based on the THR-β agonist Resmetirom has been approved by the FDA for MASH treatment [217, 384], therapeutic options remain highly limited [385]. A recent meta-analysis showed significant reduction in relative liver stiffness with combined FGF-21 analogue/THR-β agonist therapy for MASLD [386]. Clinical trial (NCT03776175), shows that co-administration of DGAT2 inhibitor with ACC inhibitors can reduce the elevation in serum triglycerides caused by ACC inhibitors [387].

Population genetics has made a huge contribution to identifying targets for the control of lipid metabolism in MASLD, expand horizons for the development of siRNA, ASO, and small molecules to suppress various targets, including PNPLA3, HSD17B13, MTARC1, GPAM, PSD3 and CIDEB [388]. However, the therapeutic effect of targeting their expression is also limited. While PNPLA3 p.I148M is a validated target, its modulation must account for its role as a risk modifier rather than a disease driver [389].

Also, it is necessary to take into account the complex and dynamic nature of the pathology. If at the initial stages of the progression of MASD the control of metabolic changes is most applicable, but then the pathology progresses, therapy should be aimed at resorption of fibrosis and prevention of hepatocyte death. A good example is the dynamics of the activity of SREBP, a key modulator of lipogenesis. As a master regulator of the DNL pathway SREBP1 exhibited a significant increase in MASLD patients [390]. However, in advanced MASH with severe fibrosis, hepatic lipid deposition often attenuates or even entirely subsides, resulting in a condition known as “burned-out MASH” with high risks of cirrhosis and hepatocarcinogenesis. The loss of SREBP and PTEN signaling [391], characteristic of burned-out MASH and HCC patients [392], promotes autophagy impairment, ER stress and other disorders in liver cells, while substantially elevating risks of carcinogenesis [393–395]. Consequently, a therapeutic intervention with significant efficacy during early progression may exacerbate the disease if utilized in late stages.

### Hepatitis

Another widespread liver disease is viral hepatitis, which includes several types, A, B, C and D. Currently, successful treatment has been achieved only for the hepatitis C virus (HCV). HCV is a major risk factor for the development ofliver cirrhosis and hepatocellular carcinoma. To date, patients are prescribed protease inhibitors, Such as sofosbuvir, simeprevir, and faldaprevir. The effectiveness of the therapy is quite high, 45–90% (varying for patients with different genotypes of the virus), but treatment is time-consuming and accompanied by adverse effects (flu-like symptoms, anemia); in addition, the production of drugs is quite expensive [396–398].

**Fig. 9:**
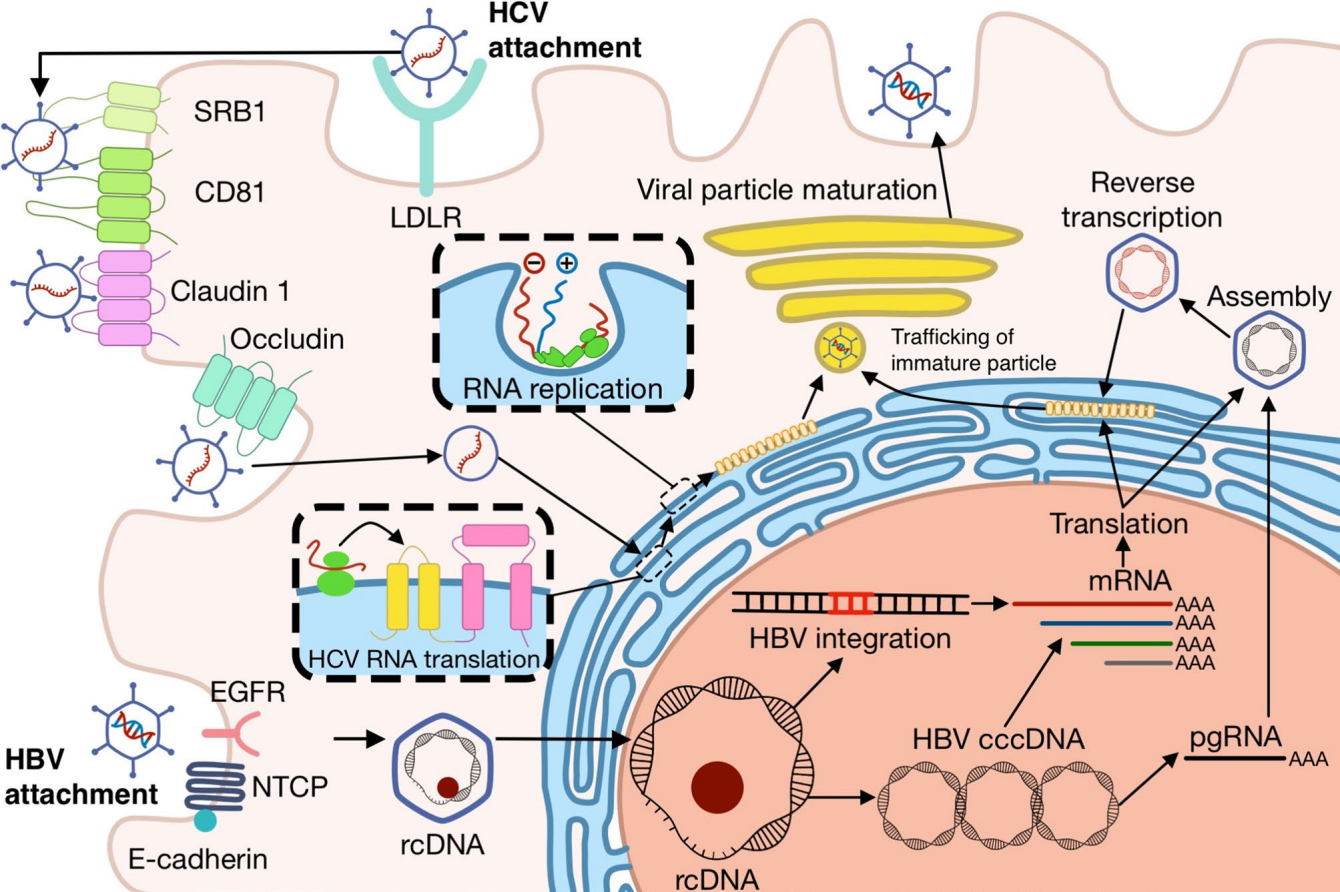
Schematic representation of the life cycle of the hepatitis C and B viruses, illustrating key steps that are targets for therapy

There is no effective treatment for hepatitis B virus (HBV). The major curing strategies include interferons and nucleosi(t)de analogues, which are not suitable for long-term use due to serious side effects. The urgent need for novel HBV treatments has driven extensive research efforts in research institutes and pharmaceutical industry [399]. New therapeutic drugs for the treatment of HBV are designed to inhibit the penetration of the virus into hepatocytes, suppressing hepatitis B surface antigen (HBsAg) secretion and transcription of the hepatitis virus, and preventing capsid formation [400]. Although the current treatment strategies efficiently suppress viral replication, the optimal endpoint of HBsAg clearance is rarely achieved. In addition, many therapeutic developments ignore the persistence of chromatin-like covalently closed circular DNA and the HBV DNA integrated in the host genome, which often reactivates the virus after discontinuation of therapy (Fig. 9) [401].

#### Inhibition of virus entry into hepatocytes

The key stage of both primary and persistent infection is the penetration of the virus into the cell. Neutralizing antibodies (NAbs) with high efficacy and high specificity have been widely used for the prevention and treatment of HBV. The majority of clinically effective NAbs target viral envelope proteins to prevent the virus from entering the host cell [402]. Recent studies demonstrated that NAbs treatment reduces circulating HBsAg and HBV DNA levels and partially restores adaptive immunity by alleviating T-cell exhaustion during chronic HBV infection [403, 404]. Despite the advantages of antibodies reducing the viral load, the mass production of polyclonal antibodies is complicated due to high costs and difficult purifying process, as well as their low stability in vivo. To solve this problem, Chen et al. [405] employed LNPs loaded with mRNA encoding three antibodies against HBsAg (G12-scFv, G12-scFv-Fc and G12-lgG); this system demonstrated a significant long-term decrease in the HBsAg levels in serum for at least 30 days after a single dose.

The use of ligands specific to receptors mediating viral penetration not only facilitates targeted drug delivery to infected cells, but also reduces viral internalization by saturating the receptor. Gao et al. [406] showed that liposomal NPs with virus-like preS/2–21 proteins attached on the surface effectively delivered encapsulated siRNA against HBV X, P, and C genes and significantly reduced the viral load and invasion in the mouse model (the levels of HBeAg, HBsAg, covalently closed circular DNA (cccDNA), and HBV DNA were reduced by 55.45%, 38.79%, 28.93%, and 24.43%, respectively). Lu et al. used the modified version of ASO Bepirovirsen (GSK3228836) conjugated with DBA-26, which competitively inhibited HBV binding to the receptor sodium/bile acid cotransporter (NTCP), effectively suppressing HbsAg [407].

As for HCV, the virus enters hepatocytes through a multi-step process that requires a number of host cellular factors (including CD81, Class B type I scavenger receptor (SRBI), claudin-1 (CLDN), occludin (OCLN), and viral envelope glycoproteins E1 and E2 [408].

DNA aptamers (small single-stranded oligonucleotides) specific for HCV E2 glycoprotein developed by Chen et al. [409] competitively inhibited the binding of E2 protein to CD81 and significantly blocked human HCV infection of hepatocyte cell cultures (Huh7.5.1) in vitro. Yang et al. [410] developed DNA aptamers specific for HCV proteins E1 and E2 that inhibited HCV infection in vitro.

Several studies have shown that siRNA against *CD81* and *SRBI* inhibits HCV penetration (> 90%) and viral load regardless of the HCV genotype [411, 412]. Suppression of *CLDN1* also inhibited HCV infection in susceptible cells (Huh7.5) [413]. The results provided by Jahan et al. [414] revealed that siRNA inhibited *CD81* and low-density lipoprotein receptor (*LDLR*), which significantly decreased the HCV viral load (by 67% and 58%, respectively). Silencing of SRBI and CLDN1 using siRNA also led to a reduction in viral load, but less efficiently. In addition, siRNA combinations siRNA-CD81 + siRNA-LDLR and siRNA-LDLR + siRNA-SRBI reduced HCV viral load by 84% compared to other siRNA combinations.

Moreover, researchers identified microRNAs that regulate the gene expression of HCV-associated receptors, preventing the virus from entering the host cells, namely, miR-548 m and miR-194 (downregulation of *CD81*) [415, 416], miR-125b (downregulation of *SR-BI*) [417], miR-182 (downregulation of *CLDN1*) [418], and miR-122 and miR-200c (downregulation of *OCLN*) [419, 420].

#### Disruption of cccDNA formation and inactivation of viral replication

None of the approved therapies such as PEG-IFN-α2a and nucleos(t)ide analogues affect the cccDNA of HBV, which exists episomally and is responsible for viral persistence and reactivation [421]. It was reported that only several copies of cccDNA may cause the infection to recur after the treatment is discontinued [422].

CRISPR/Cas is widely used to identify the host factors involved in various stages of infection for highly sensitive detection and inactivation of viral nucleic acids in a cell [423–425]. Several CRISPR/Cas systems have been designed for inactivating hepatitis B viral genes to target conserved regions of cccDNA in vitro [426–428] and in vivo [426, 429–432]. In vitro experiments with the CRISPR/Cas9 system in synergy with nucleos(t)ide analogues show a significant functional inactivation of cccDNA, suppressing the viral replication [433]. In 2014, CRISPR/Cas9 was first used to suppress viral hepatitis in vitro, which inhibited HBV infections up to 8-fold [426]. Several works [434–438] demonstrated the advantages of a combination of gRNAs targeting conserved regions of the HBV genome compared to application of a single gRNA for CRISPR/Cas9 therapy. These gRNAs inhibited extracellular HBsAg and HBeAg, reducing the level of total HBV DNA and cccDNA without affecting the cell viability. Fei et al. [434] highlighted that a combination of gRNA reduced cccDNA by almost 50% better compared to each gRNA used separately. However, this approach has limitations, including insufficient stability of paired gRNAs [439]. Zeng et al.[436] used modified extracellular vesicles as a carrier for the delivery of Cas9/gRNA ribonucleoprotein, which decreased the extracellular levels of HBeAg, HBsAg, and HBV DNA and the intracellular levels of HBcAg and cccDNA both in vitro and in transgenic HBV mice.

Kumar et al. [440] suggested using a combination of gRNA and RNA encoding BE, and thus, various BE inactivated conservative HBV sites without the risk of forming transcriptionally active elements, as in the case of cutting out a section of viral DNA [427, 441, 442]. The study [443] confirmed the effectiveness of this approach, demonstrating that a combination of BE and nucleos(t)ide analogues efficiently eliminated the virus cccDNA in primary hepatocyte culture and in differentiated hepatocytes (HepG2-NTCP cells).

Recently, a new potential therapeutic approach for cleavage of HBV cccDNA using an engineered ARCUS nuclease (ARCUS-POL) was described [444]. Systemic injection of LNP-encapsulated ARCUS-POL mRNA into mice and non-human primates decreased the episomal vector AAV containing a part of the HBV genome that served as a surrogate for cccDNA. Moreover, circulating surface antigen was steadily reduced by 96% in mice. Notably, LNP-based therapy PBGENE-HBV (Precision BioSciences) [445] with encapsulated mRNA ARCUS nuclease targeting highly conserved regions of cccDNA and integrated HBV DNA is currently evaluated in phase 1 clinical trials (NCT06680232) [446].

Recent data indicate that additive modulation of host DNA damage repair pathways and cell cycle proteins expression enhances the antiviral effect of CRISPR/Cas9 and significantly reduces the intracellular HBV pregenomic RNA (pgRNA) [447, 448], thus, making many factors of the DNA damage repair system a target for therapeutic intervention. Upregulated proliferating cell nuclear antigen pseudogene 1 (*PCNAP1*) and proliferating cell nuclear antigen (*PCNA*) mRNA are observed in the liver of patients with HBV, making these genes a possible target for therapy. Considering the regulatory axis PCNAP1-miR-154-PCNA, Feng et al. [449] showed that downregulation of miR-154 (mediated by lncRNA PCNAP1) activates PCNA, promoting HBV replication and cccDNA accumulation. The cytokinesis 11 dedicator (DOCK11) is also considered a promising target, since it participates in the conversion of rcDNA into HBV cccDNA with a subsequent activation of DNA damage repair systems. DOCK11 also controls retrograde HBV traffic to the nucleus, because it is associated with Arf GAP with GTP-binding protein-like domain, Ankyrin repeat, and PH domain 2 (AGAP2) [450]. Okada et al. [451] inhibited DOCK11 using the LNP made of Dlin-MC3-DMA (ionizable lipid), DSPC, PEG2000-C-DMG, and cholesterol loaded with modified siRNA targeting *DOCK11*. The inhibition of DOCK11 reduced the cccDNA by more than 50% in the HBV mouse model.

The serine/threonine-protein kinase proto-oncogene (PLK1), a regulator of the cell cycle and centrosome formation, is a proviral HBV replication factor. Treatment with PLK1 inhibitors had a suppressive effect on the HBV replication [452]. Foca et al. [453] developed a siRNA targeted at *PLK1* encapsulated in LNP for anti-HBV treatment, which demonstrated a strong and specific antiviral activity in HBV-infected hepatocytes. The achieved inhibition efficiency of the main markers of HBV replication (HBeAg/HBsAg, HBV RNA, and HBV DNA) was comparable to that during treatment with the clinically approved antiviral drug Tenofovir (TFV) and siRNA targeting HBV.

**Fig. 10:**
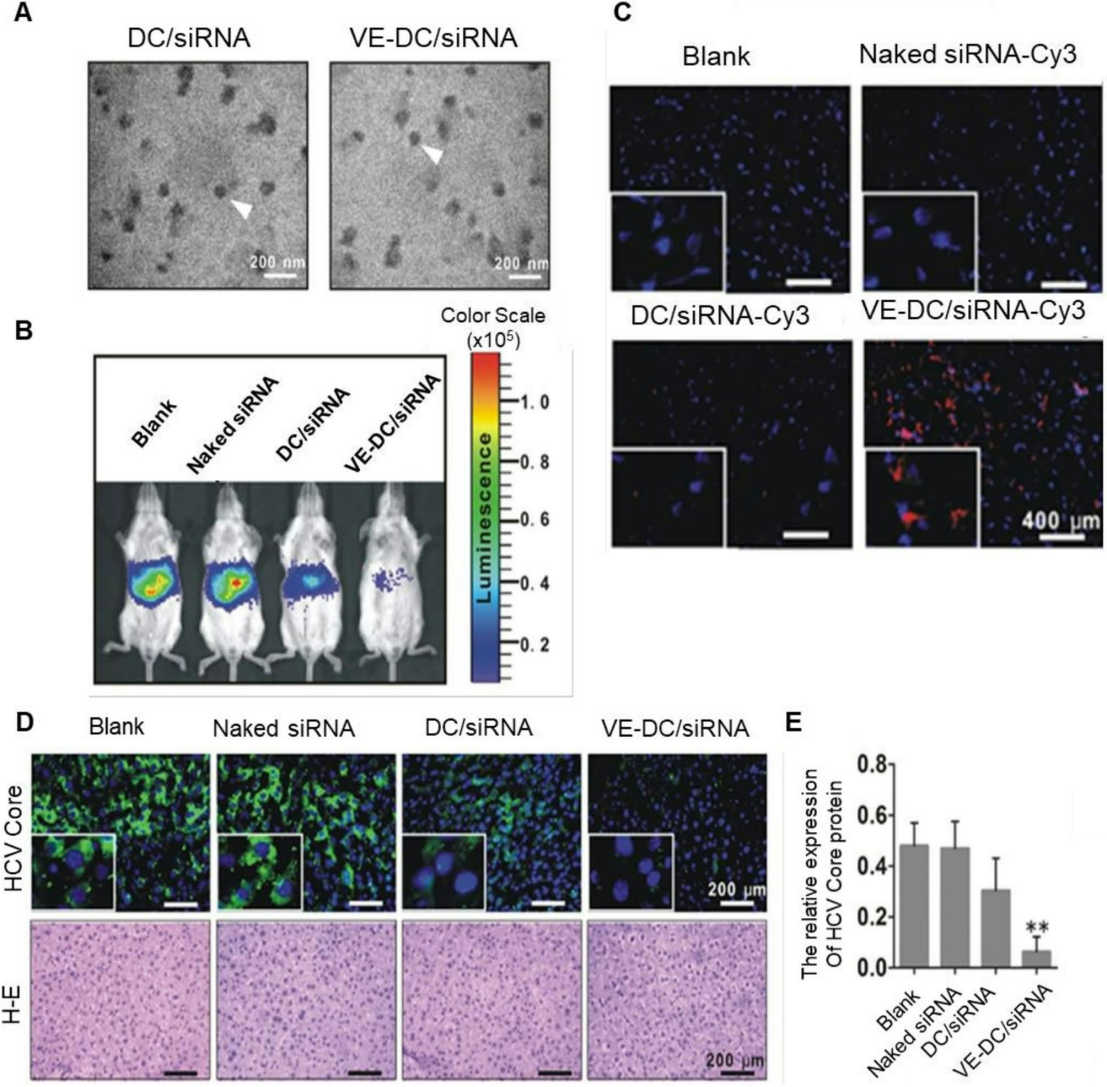
Targeted silencing of HCV core protein via VE-decorated dendrimeric siRNA delivery: **A** Transmission electron micrographs of DC/siRNA and VE-DC/siRNA. White scale bars are 200 nm. **B** Representative whole-body images for luciferase expression in BALB/c mice hydrodynamically injected with the luciferase expression plasmid pGL3-5′UTR-luc and 24 h later treated intravenously with and without naked siRNA, DC/siRNA, or VE-DC/siRNA (siRNA-5′UTR). **C** Representative fluorescent images of the liver distribution of Cy3 signal in mice 1 h after the injection of naked siRNA-Cy3, DC/siRNA-Cy3, and VE-DC/siRNA-Cy3. The nuclei were stained with DAPI (blue). White scale bars are 400 μm. **D** Representative immunofluorescent staining result for liver-specific expression of the core protein in mice treatment. Sections of liver tissue were stained with the antibody against the core protein, and then, FITC-labeled secondary antibody was applied (green fluorescence). The nuclei were counterstained with DAPI (blue). White scale bars are 200 μm. Representative images of histological sections of liver tissue. **E** The relative expression of the core protein is quantified by core/β-actin densitometric ratio in treated mice. **p < 0.01, VE-DC/siRNA vs. Blank. Reproduced under terms of the CC-BY license [456]. 2016, Liang Duan et al., published by Springer Nature.

Since HCV does not integrate into the genome of an infected cell or form stable epistomal elements, it can be effectively eliminated by blocking viral replication [454]. Moon et al.[455] developed lipidoid NPs containing siRNA targeting the protein kinase C-related kinase 2 (*PRK2*), which inhibited the NS5B RNA-dependent RNA polymerase (RdRp) activity and decreased the HCV genome abundance. Duan et al.[456] delivered three siRNAs (sin5x, sin 4 A, sin 5 A) targeting key components of the HCV replication complex [457] using liposomes-vitamin E and cholesterol-based cationic liposomes. As a result, this platform efficiently suppressed the gene and protein expression of HCV (Fig. 10).

**Fig. 11:**
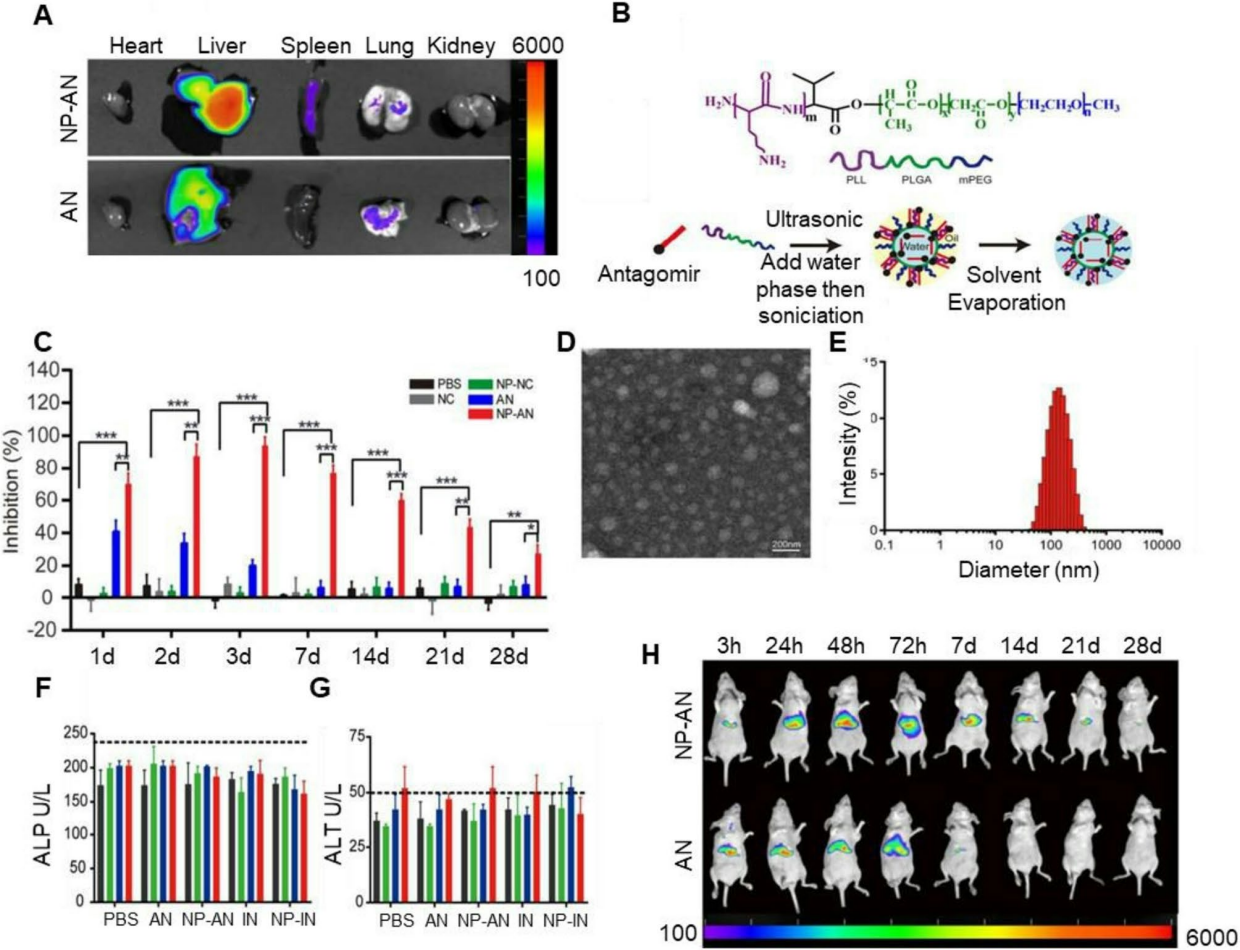
Efficient and long-term miR-122 inhibition by mPEG-PLGA-PLL nanoparticle-mediated delivery of antagomirs: **A** Fluorescence images of the distribution of mPEG-PLGA-PLL NPs in the organs of mice. Fluorescence results showed that NPs-AN were retained in the Liver for over 28 days and the pure AN only for 3 days. **B** Preparation and characterization of mPEG-PLGA-PLL NPs. **C** MiR-122 inhibition measured for mice treated with different ANs, negative control (NC), negative control-loaded NPs (NPs-NC), and NPs-AN. The NPs-AN showed the highest inhibition of miR-122 on the 3rd day, which gradually decreased the following days. On the 28th day, the inhibition rate Maintained at over 20%. The AN inhibition ability only lasted for 3 days. **D** TEM of antagomir-loaded NPs. **E** The size distribution of the used NPs, with an average size of approximately 150 nm. **F** All the groups showed no negative influence on ALP level. **G** Serum ALT of mice injected with NPs for different times. All the groups showed no negative influence on ALT level. **H** Fluorescence images of mice administered with NPs. The fluorescence results showed that NPs-AN were retained in the Liver for over 28 days, while the pure AN, only for 3 days. Reproduced with permission [466]. 2018, published by Royal Society of Chemistry.

Several microRNA (miR-196, miR-296, miR-351, miR-431,miR-199a, let-7,miR-181c, and miR-448) are able to inhibit the HCV replication by directly interacting with the HCV genome regions and HCV NS5A gene [458, 459]. However, in contrast, some microRNA can promote HCV replication. The best-studied microRNA that promotes the replication of the HCV is miR-122. MiR-122 in association with human Argonaute proteins (Ago 1 to 4) binds to the HCV genome, promoting viral RNA accumulation [460]. While the precise mechanism of HCV replication promotion remains incompletely understood, different roles of miR-122 have been identified, including translation stimulation [461], genome stabilization, and directly contributing to the genome amplification [462]. MiR-122 antagonists have shown promising results in a prolonged reduction of viral load in both preclinical and clinical trials [463–465]. Fu et al. [466] reduced miR-122 by more than 90% 72 h after the injection of mPEG-b-PLGA-b-PLL nanoparticulate platform delivering miR-122 antagomir. The inhibition of miR-122 lasted for 28 days with a limited dosage in vivo. (Fig. 11).

Therapeutics Miravirsen (SPC3649) is an LNA-modified phosphorothioate oligonucleotide inhibiting miR-122is undergoing phase IIa clinical trials (NCT01200420); however, the clinical trial of Miravirsen encountered serious immune-mediated adverse events [2]. The recent emergence of resistant HCV mutants [467] suggests that the virus can adapt to grow in the absence of miR-122. Similar drug RG-101 (antagonises miR-122) is undergoing phase Ib of a clinical trial; it was reported to reduce viral load in patients with HCV, but RG-101 was discontinued after the drug was found to cause hyperbilirubinemia [2]. The above-mentioned contradictory results underscore the importance of organ- and cell-specific targeting.

#### Suppression of hepatitis virustranslation

All HBV transcripts, including subgenomic ones and pgRNA, share an identical 3’ end, which becomes a potential target for the simultaneous suppression of all viral transcripts via siRNA-mediated inhibition and ASO-induced degradation [401, 468, 469]. Several siRNAs were tested in animal models, and some of them have progressed to clinical trials (Table 3). Most of them were developed for subcutaneous injections (AB-729, VIR-2218, JNJ-3989, ARC-521, bepirovirsen, and RG6346) and for intravenous administration (ARC-520, ARB-1467, and ARC-521). However, the use of these candidates requires repeated dosing, sometimes in combination with other treatments such as PEGylated IFN and administration of nucleosi(t)de analogues [470].

**Table 3:** Clinical trials of gene therapeutics for hepatitis treatment

Inventor	Product	Delivery system	Target	Status	Clinical trial	Results
Arrowhead Pharmaceuticals	ARC-520/ARC-521	Dynamic PolyConjugate (DPC)	All HBV RNAs	Terminated	NCT02452528	Up to 2.6 log IU/mL HBsAg reduction in 2 patients and HBsAg seroclearance in 1 patient (HBeAg positive patients); 0.4 log IU/mL HBsAg reduction in 4 patients and HBsAg seroclearance in 1 patient; Suppression was maintained up to > 85 days
					NCT02577029	
					NCT02604212	
					NCT02065336	
Arrowhead and Janssen	JNJ-3989	TRiM™ GalNAc conjugation	All HBV RNAs	Phase I, IIa	NCT04535544	Up to 2.6 log IU/mL HBsAg reduction; Suppression was Maintained up to 113 days
					NCT05275023	
					NCT04667104	
					NCT04439539	
Arbutus Biopharma	ARB-1467	LNP	All HBV RNAs	Phase IIa	NCT02631096	Maximum individual HBsAg reduction of 2.7 log IU/mL; Suppression was Maintained up to 70 days
Aligos Therapeutics	ALG-125755	GalNAc conjugation	HBV S region	Phase I (Discontinued)	NCT05561530	Up to 1.5 log IU/mL HBsAg reduction in mice
Alnylam Pharmaceuticals	VIR-2218	GalNAc conjugation siRNA ESC +	HBV X region	Phase II	NCT04507269	Up to 2.55 log IU/mL HBsAg reduction; Suppression was Maintained up to 336 days
					NCT05484206	
					NCT06650852	
					NCT03672188	
					NCT06491563	
					NCT02826018	
Arbutus Biopharma	AB-729	GalNAc conjugation	All HBV RNAs	Phase IIa	NCT04980482	Up to 2.16 log IU/mL HBsAg reduction; Suppression was Maintained up to 336 days
					NCT04820686	
					NCT04847440	
Dicerna Pharmaceuticals	RG-6346	GalXC GalNAc conjugation	HBV S region	Phase Ib, IIa	NCT03772249	Up to 1.87 log IU/mL HBsAg reduction; Suppression was Maintained up to 448 days
					NCT04225715	
					NCT04225715	
Suzhou Ribo Life Science	RBD-1016	RIBO-GalSTAR™ GalNAc conjugation	HBV X region	Phase II	NCT06649266	Up to 1.26 log IU/mL HBsAg reduction; Suppression was Maintained up to 168 days
					NCT05961098	
Sino Biopharm	TQA3038	GalNAc conjugation	All HBV RNAs	Phase Ib/IIa	NCT06085053	Data not available
					NCT06452693	
Jiangsu Hengrui Pharmaceuticals Co.	HRS-5635	GalNAc conjugation	Data not available	Phase II	NCT06425341	Data not available
					NCT05808374	
Staidson	STSG-0002	Data not available	Data not available	Phase II Terminated	NCT05760703	Data not available
					NCT05760781	
Suzhou Xingyao Kunze Biopharmaceutical Co	HT-101	GalNAc conjugation	HBV S region	Phase II	ChiCTR2200066547	greatest mean HBsAg reduction reached 3.29 log; Suppression was Maintained up to 336 days
					CTR20244730	
					NCT06746311	

The first anti-HBV RNAi-based therapeutic agent to be administered to humans was ARC-520, which contains two different siRNAs and a polymer-based excipient (ARC-EX1), which enhances endosomal release into the cytoplasm [471]. However, clinical studies of ARC-520 failed (RO 7062931), demonstrating important limitations of the platform in addition to drug-induced mortality in primates. According to the mathematical model [472], the effectiveness of ARC-520 in reducing HBsAg and HBeAg was 96% on the first day and decreased to 50% over the next 1.5–3 months. Moreover, HBsAg reduction was significantly less pronounced in HBeAg-negative individuals, which may be explained by the predisposition of ARC-520 to target cccDNA-derived transcripts, rather than integrated HBV [473–475]. Thus, the search for new therapeutic siRNAs continues [476].

The duration of effective suppression of HBsAg was significantly increased with the use of VIR-2218 and JNJ-3989, based on modernized siRNA platforms ESC + and TRiM™, respectively. VIR-2218 provided a sustained decrease in HBsAg for 48 weeks after treatment [477]. JNJ-3989 provided even more favorable results, with 81.5% of patients maintaining a reduced HBsAg response 48 weeks after treatment [478]. RBD-1016 also uses a modified delivery approach RIBO-GalSTAR™ developed by Ribo Life Science, based on GalNAc (N-Acetylgalactosamine) -siRNA mechanical coupling. RBD-1016 has entered phase II of clinical trials (NCT06649266) and showed excellent and sustained efficacy in reducing HbsAg [479]. The GalXC platform (RG6346) uses DsiRNA with four-base hairpin loop modified by GalNAc Sugars instead of the usual triantennary GalNAc for conjugation with siRNA, which highly efficiently targets the liver with a long-lasting effectiveness in clinical trials, up to 1 year [480].

A notable result was achieved in a complex therapy consisting of siRNA JNJ-3989, core protein allosteric modulators (CpAM), and nucleose(t)id analogues. Among 12 people with chronic HBV, the treatment resulted in a significant decrease of viral markers such as HBsAg, HBV DNA, RNA, and HBcrAg by day 113 of therapy. This combined therapy was well tolerated without serious side effects [481]. Notably, a recent clinical trial (NCT04225715) also studied the effectiveness of combination therapy: it included nine separate testing groups, each treated with a unique combination of therapeutic agents. Seven of these testing groups included siRNA RO7445482 as one of the therapy components combined with nucleos(t)ide analogues, CpAM, TLR7 agonists, PEG-IFN, and programmed cell death 1 Ligand 1 (PD-L1) LNA [482]. Researchers have also explored the combined application of siRNA ALG-125903 with ASO ALG-020579, alongside other anti-HBV agents such as nucleos(t)ide analogues and CpAM, which rapidly and effectively suppressed HBsAg in vivo [483].

ASOs for HBV therapy are also being actively developed. Bepirovirsen (previously ISIS 505358; GSK3228836) [484] is an ASO using 2’-O-methoxyethyl-modified ribonucleotides and modified phosphorothioate linkages in gapmer design to increase stability in vivo. Aside from its primary effect through RNAse H1 activity, Bepirovirsen can mediate TLR-9 activity, thereby facilitating the activation of the immune response to the HBV (due to CpG Class II motif recognition) [485, 486]. Currently, Bepirovirsen is the only oligonucleotide drug against HBV that has entered phase 3 clinical trials (NCT05630820); the results demonstrated dose-dependent decrease in HBsAg and HBV DNA after 4 weeks of treatment with a favorable safety profile [486, 487]. An alternative GalNAc-conjugated version of Bepirovirsen (GSK3389404) has been tested in a phase II clinical trial (NCT03020745). However, the efficacy of GSK3389404 was weaker than that of unmodified Bepirovirsen[488] because of low drug accumulation in non-parenchymal cells (such as KCs or LSECs), which reduced the TLR activation and overall antiviral activity [486].

Alternative approaches based on vectors carrying the primary-miR sequence are currently being actively studied. Primary-miR-31 targeting the HBx region was delivered using recombinant AAV, suppressing viral markers for at least 32 weeks without signs of toxicity or immunostimulation [489]. Additionally, miR-135 delivered with AAV vector reduced HBsAg and HBeAg in HBV transgenic mice, maintaining low levels of these antigens for up to 15 months [490]. Similarly, Ivacik et al. [491] used a lentiviral delivery system and demonstrated the effectiveness of pri-miR-31, achieving sustained inhibition of HBV replication without any signs of hepatotoxicity in mouse model.

An intriguing approach combining CRISPR/Cas9 and RNA interference used a triple cassette, gRNA-microRNA-gRNA, controlled by a single U6 promoter. pri-microRNA processing enzymes Drosha/DGCR8 cleave and generate two guide RNAs and pri-miR-31 for synergistic suppression of HBV. This strategy effectively suppressed HBV replication in vitro and in vivo and additionally disrupted HBV genomic DNA and cccDNA in vitro [492].

The CRISPR/Cas approach is also used for HCV therapy. In a recent study, CRISPR/Cas13b targeted a highly conserved internal ribosomal entry site (IRES) HCV RNA, reducing HCV replication and viral protein translation in vitro [493]. The CRISPR/FnCas9 also suppressed HCV replication and viral protein translation, inhibiting the virus by more than 50%[494].

#### Capsid formation and virion secretion

During HBV infection, subviral particles (SVP) are produced in large excess, depleting virus-neutralizing antibodies and reactive immune cells [495, 496]. Nucleic acid polymers (NAP, REP 2139) block the release of HBsAg, thereby preventing the assembly of HBV subviral particles [497, 498]. Importantly, combination therapy using REP 2139 and PEG-IFN is now undergoing phase 2 trial (NCT02233075). This combination is easily tolerated and safe, and provides Functional control of HBV and HDV co-infection in patients 1 year after therapy [499].

The HCV uses the biosynthetic pathway of very low-density lipoproteins (VLDL) to assemble and release viral particles from cells, which was comprehensively reviewed in [454, 500]. Therefore, regulation of lipid metabolism may help in the treatment of HCV. Recent study [501] has shown that inhibition of the transmembrane 6 Superfamily member 2 (*TM6SF2*) (lipid metabolism and HCV secretion pathway) using siRNA suppressed the secretion of HBV, HCV, and HDV virions. In addition, inhibition of the TM6SF2 reduces HCV secretion (by 50%) and HCV viral load in vitro [502]. Although therapy based on TM6SF2 inhibition is associated with the development of liver steatosis, a limited-duration treatment (e.g., a few months) is unlikely to cause adverse long-term health impacts such as hepatic steatosis. Lee et al. [503] also showed that miR-99a regulates lipid metabolism by regulating mTOR (mammalian target of rapamycin) expression, which violates the replication and packaging of HCV.

#### Modification of T-lymphocytes as a strategy for suppressing HBV and HCV infections

Functional treatment using new antiviral drugs is achieved Mainly in patients with low HBsAg levels and, consequently, in the immune control phase. Due to this, in most phase 2 trials of combination therapy against HBV, including siRNA or ASO with pharmacological HBV inhibitors (nucleos(t)ide analogues, CpAM, TLR7 agonist, and PEG-IFN), large proportion of patients experienced viral relapse after therapy. Thus, the lack of powerful components restoring immune depletion in new combinations against HBV may hinder their long-term effectiveness [475, 504].

Chronic infection leads to depletion or anergy of CD4 + and CD8 + T cells. Depleted CD4 + and CD8 + T cells are no longer effectively stimulated by their respective ligands, and there are elevated levels of co-inhibitory receptors, programmed death factor-1 (PD1), and pro-apoptotic molecules [505].

Zai et al.[506] successfully suppressed HBV by co-delivery of siRNA-HBV (with a genotypic coverage of 98.55%) and mouse IL-2 mRNA encapsulated within an optimized LNPs (tLNPs), which enables synergistic antigenic and immune control of HBV, triggering strong HBV-specific CD4 + and CD8 + T cell responses by expressed mIL-2 protein. The expression of viral antigens and DNA significantly reduced (up to 3log10 reduction; vs. PBS) in dose- and time-dependent manners after single or multiple dosing, with satisfactory safety profiles.

T cell engineering is a revolutionary strategy for restoring the function of HBV-specific T cells. A recent systematic review by Elena Morte-Romea [507] covered 7 preclinical studies on the development of TCR for HBV therapy [508–514]. In addition, participants are currently being recruited for at least four clinical trials (NCT04745403, NCT05195294, NCT06617000, and NCT05417932) to study the safety and efficacy of HBV-specific TCR-engineered T cells in patients with HBV-related HCC. Furthermore, in the work of Preece et al. [515], HBV-specific T cells were engineered to express the recombinant T cell receptor (rTCR), and endogenous T cell receptors (eTCR) were reduced using the new CRISPR-STOP technology, which introduced premature stop codons into eTCR. eTCR destruction was associated with a higher expression of the introduced rTCR. We found only one publication related to HCV infections, in which the developed CAR-T cells targeted HCV E2 glycoprotein to eliminate HCV-infected hepatocytes in vitro [516].

Some T-cell factors may interfere with the control of chronic HBV infection, for example, PD-1. PD-1 inhibitors and targeted therapy against HBV can act synergistically, suppressing HBV gene expression and significantly increasing the survival of HBV transgenic mice [517, 518]. In an HBV mouse model [519], siRNA targeting *PD-L1* increased the functionality of HBV-specific CD8 + T cells after therapeutic vaccination and allowed for a more stable antiviral effect in the liver. Dolina et al. [520] showed that PD-L1-siRNA encapsulated in lipidoid NPs administered into mice accumulate in KCs, silencing *PD-L1*, increasing the intrahepatic accumulation of NK and CD8 + T cells in the liver, and improving clearance of infected cells. Phase II clinical trials (NCT04225715) are currently underway employing a combination of LNA-ASO RO7191863 (targeting PD-L1), siRNA RO7445482 (mentioned earlier), and pharmacological HBV inhibitors. Oligo Therapeutics Inc. also announced the development of ALG-072571, GalNAc PD-L1-siRNA, which has demonstrated potential for restoring immune responses against HBV and consequent clearance of HBV infection [521]. However, there are concerning reports of immune-mediated liver injury caused by immune checkpoint inhibitors (ILICI), discussed in detail in a recent review by Triantafyllou et al. [522] Exposure to immune checkpoint inhibitors (such as PD-L1 inhibitors) can promote the activation of self-reactive T cells and T cell diversification and expansion following treatment, in addition to B cell dysregulation and increased production of autoantibodies, which disrupts the immune tolerance of the liver and renders it more susceptible to sterile inflammation.

#### Discussion: current limitations and research directions of hepatitis treatment

The current strategies based on siRNAs target conserved sequences in the overlapping open reading frames (ORFs) of the HBV genome. These regions show high homology (≥ 90%) across different genotypes of the virus. As a result, siRNA drugs often target the S and X reading frames of the hepatitis B virus [523]. But the challenge in developing antiviral genomic medicine-based therapeutics is that viruses mutate quite rapidly and generate high heterogeneity of the genome (i.e., HBV quasispecies), which often allows them to alter the target sequences of siRNAs and gRNAs and significantly reduce or abolish the effectiveness of such therapy [425, 524–527]. Numerous studies have shown that targeting highly conserved regions of viral genomes can effectively suppress the replication of various viruses, including HCV [528, 529] and HBV [530]. However, data from both mouse models and clinical studies [506, 531] suggest that siRNA monotherapy is insufficient to achieve complete elimination of HBV cccDNA or integrated HBV DNA [532] and minimal effect on restoring HBV-specific immunity [533].

Given these limitations, CRISPR/Cas9 offers a more direct alternative by targeting the viral genome. Nevertheless, single gRNA for Cas9 cleavage may not be sufficient to completely inactivate HBV cccDNA [426, 433], and it is preferable to use a multilocus targeting approach. Multilocus targeting not only induces multiple site mutations but can also cause large deletions between target loci and thus increase the likelihood of viral genomic disruption [534]. The multiplexing strategy was also effective for treating diverse HBV genotypes from different geographic regions [535]. Several research groups [441, 536] reported that dual gRNA guided CRISPR/Cas9 system can significantly reduce the levels of HBV products. However, multilocus targeting has disadvantages, including generation of transcriptionally active episomal variants and difficult comled removement of integrated HBV DNA sequence without affecting the host sequence [537]. As discussed previously, a promising solution is the use of BEs that induce multiple stop and nonsense codons leading to loss of functionality of persistent viral genomes [538].

Theoretically, even a single copy of replication-competent cccDNA can lead to resumption of viremia after cessation of antiviral therapy. Thus, sustained suppression of HBV replication usually requires combination therapy, which should probably include immunomodulators to restore host immunity. Considering that immunotherapy has become the mainstream of modern HBV research, the combination of siRNA or CRISPR with immunomodulators is considered a promising avenue to improve the functional cure rate [482]. However, as mentioned earlier, the use of immunotherapeutic drugs, despite their promising prospects, requires constant monitoring for the development of side effects such as ILICI [539].

### Fibrosis

**Fig. 12:**
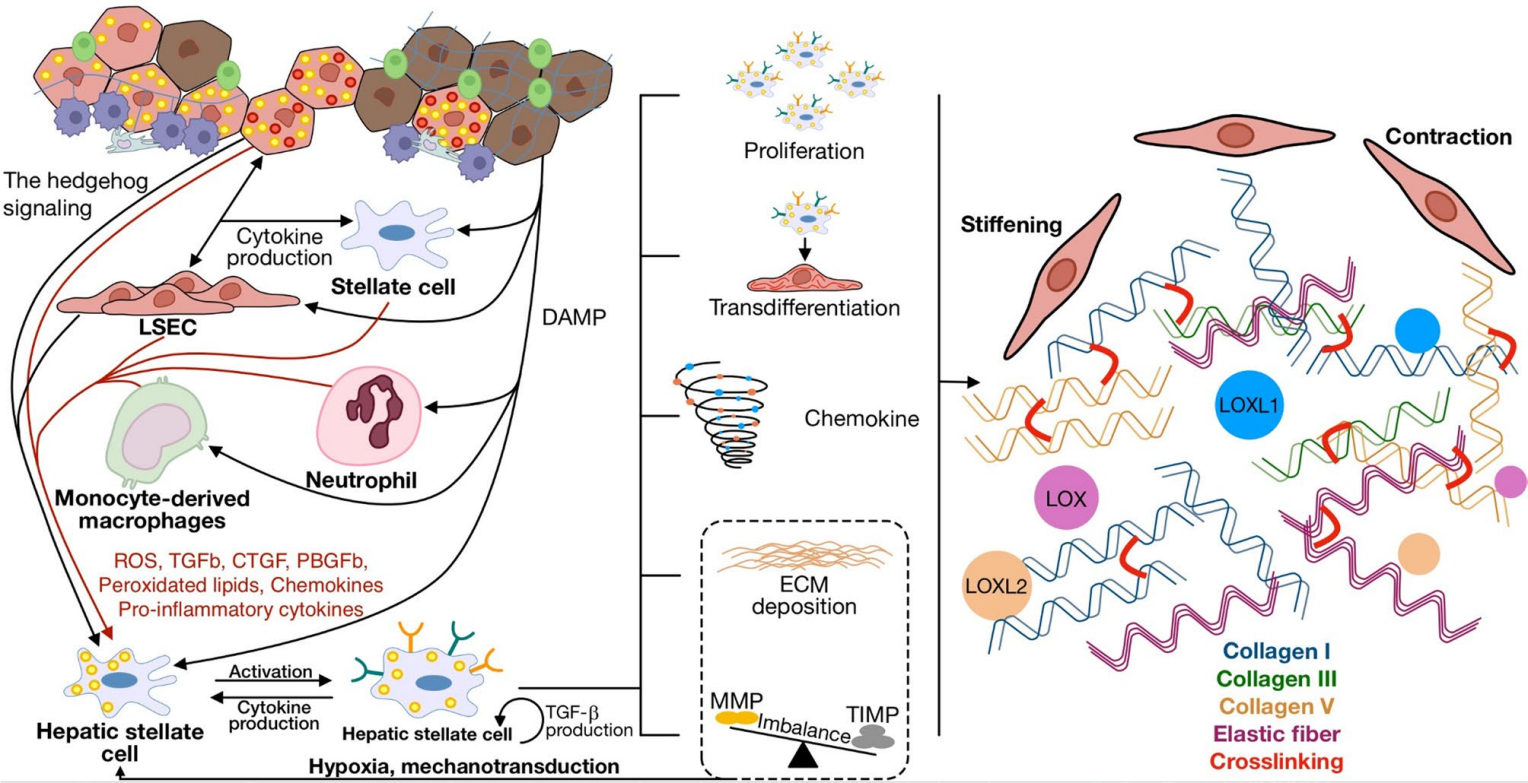
Pathophysiological mechanisms of fibrosis development. Development of liver pathologies is associated with damage to hepatocytes, release of DAMP, proinflammatory mediators, and disruption of signal cascades. This activates HSCs, which begin to secrete cytokines that support their proliferation, activation, migration, and transdifferentiation into myofibroblasts. An increase in the number of myofibroblasts, a shift in the balance between MMP and TIMPs, and increased production of ECM components leads to excessive deposition of ECM, increasing tissue stiffness and causing the development of hypoxia, which, in turn, also contribute to cellular stress and aggravate fibrosis

Liver fibrosis, which can progress to cirrhosis in its advanced stages, develops in response to chronic liver damage of various origins. Fibrosis is characterized by excessive formation and deposition of ECM, including collagen [540], accompanied by a disruption of tissue architecture, development of portal hypertension, and cellular hypoxia. In the case of chronic damage, the regenerative process is disrupted, and hepatocytes are replaced by ECM proteins (Fig. 12) [541, 542]. Fibrosis is a progressive pathological process associated with various liver diseases, including NASH and hepatitis. A key event in liver fibrosis is the activation of HSCs: as a result, HSCs acquire a myofibroblast-like phenotype characterized by increased proliferation, loss of vitamin A stores, and upregulation of α-SMA expression and collagen (types I and III) into the extracellular space [543]. HSCs are recognized as the most effective target for the development of antifibrotic drugs due to their critical role in the initiation and progression of liver fibrosis. Activated HSCs continuously produce growth factors, fibrogenic cytokines, and chemokines, which leads to the progression of inflammation. Among these, TGF-ß directly promotes fibrogenesis by inducing the transcription of types I and III collagen through the small mothers against the decapentaplegic (Smad) signaling pathway. Moreover, TGF-ß influences the activated HSCs themselves, providing their survival and stability [544].

There are two main approaches to antifibrotic therapy: (i) inhibition of HSCs’ activity coupled with treatment of inflammation, and (ii) resorption of fibrous tissue with restoration of tissue structure [545, 546]. This classification is quite arbitrary, as these mechanisms are highly interrelated.

Conventional pharmacological approaches for fibrosis therapy remain highly limited in addressing fibrosis itself, particularly when using monotherapy [547]. For example, the recently approved drug resmetirom provided improvement in fibrosis in only 24–26% of study participants[548], while another study reported high rates of drug discontinuation due to adverse events and the need for long-term follow-up in patients taking resmetir [549]. Ursodeoxycholic acid, a widely used Hepatoprotective agent, reduced fibrosis progression risk by 76% at 4 years and 61% at 8 years, but but didn’t affect on regression of fibrosis among patients with primary biliary cholangitis [550]. In addition, its use improved liver biochemical markers in patients with NASH, but did not lead to histological improvement [551, 552]. Similarly, obeticholic acid, which entered phase III clinical trial (NCT02548351) for the treatment of NASH, confirmed its efficacy in preliminary results [553], but in some cases obeticholic acid treatment provoked liver fibrosis and chronic liver failure among many other serious side effects [554].

Given limitations of therapies targeting primary pathology, researchersare exploring new antifibrotic agents that directly target specific pro-mediators. These include pirfenidone (NCT01046474, NCT05542615) and simtuzumab (NCT02466516, NCT01672853), which have recently entered phase 2 clinical trials. Pirfenidone is a non-peptide synthetic chemical that inhibits the production of TGF-β1, TNF-α, PDGF, IL-1β, and COL1A1 [555]. Despite the encouraging results of the ODISEA study [556], additional evaluations are needed [557]. Simtuzumab is a humanized monoclonal antibody against lysyl oxidase homolog 2 (LOXL2), an enzyme involved in collagen crosslinking. In trials of patients with bridging liver fibrosis or compensated cirrhosis associated with NASH, simtuzumab showed neither a decreasing hepatic collagen content nor hepatic hemodynamics improvement [558].

Thus, new drugs must target multiple pathways. The first strategy can be achieved by modulating inflammatory signaling pathways associated with HSC activation. For instance, a number of studies revealed a link between inflammation, ER stress, oxidative stress, hypoxia, and activation of HSCs, largely due to the Notch pathway activation [559, 560]. Yu et al. [561] revealed interplay between inflammation and Notch mediated fibrogenesis due to TLR4 activation and NF-KB-mediated overexpression of Jag1 (Notch1 ligand). They also showed that the *Jag1*-targeted GalNAc-ASO effectively reverses NASH-induced fibrosis. Similarly, Maradana et al. inhibited the HSCs activation using vitamin A-coupled liposomes with plasmids expressing shRNA (VA-lip-TLR4-shRNA), which silenced TLR4, slowing down the ECM deposition in the liver tissue [562].

**Fig. 13:**
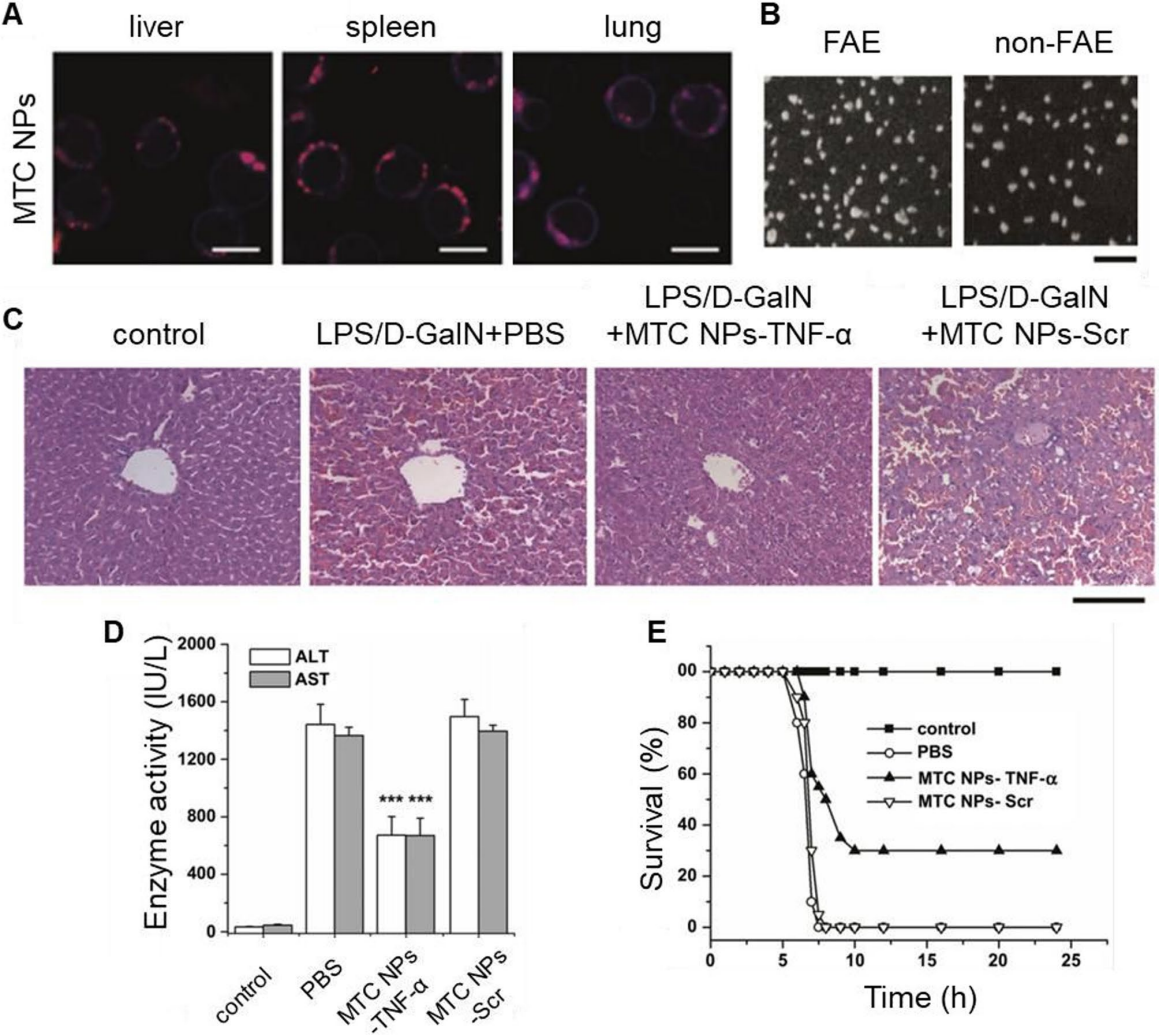
Oral delivery of TNF-α-siRNA via MTC-modified NPs alleviates hepatic inflammation and fibrosis: **A** Representative CLSM images showing TAMRA-siRNA (red) distribution in macrophages from the mouse liver, spleen, and Lung. Scale bar is 10 mm. **B** Scanning electron microscopy images of permeated MTC NPs collected from the basolateral side of follicle-associated epithelium (FAE) and non-FAE. Scale bar is 1 mm. **C** Hematoxylin- and eosin-stained Liver sections from mice 6 h after LPS/D-GalN stimulation. Scale bar is 1 mm. **D** ALT and AST levels in mice 6 h after LPS/D-GalN stimulation. **E** Survival percentage of mice after LPS/D-GalN stimulation. Reproduced with permission [369]. 2013, published by Elsevier.

Hypoxia-inducible factors (HIFs) are also involved in the progression of liver fibrosis [563–565]. HIF-1a promotes HSCs activation and fibrosis primarily by activating the PTEN/p65 signaling pathway and also by modulating Notch signaling [566–568]. Lyu et al. [569] delivered HIF-1a-siRNA in combination with silibinin (SLB) using LNP carriers (SLB-HIF1a-siRNA-LNPs) to the liver of rats with fibrosis. SLB-HIF1a-siRNA-LNP suppressed the HIF-1a-associated pathway, significantly reducing the inflammation and fibrosis in the liver. Liu et al. [570] used VA-PEG-modified carbon nitride-based nanosheets loaded with HIF-1a-siRNA to downregulate HIF-1α expression in HSCs, which improved the hypoxic microenvironment in the fibrotic liver and contributed to hepatocyte recovery. HIF-2α is also involved in liver fibrosis through the regulation of various molecular cascades [565, 571]. Yan et al. [572] used shRNA to reverse the HIF-2α-induced activation of the Hippo signaling pathway and glutaminolysis, thereby reducing HSC activation markers. Zhang et al.[573] used mRNA delivery for in vivo production of an IL-11 single-chain variable fragment (scFv), which neutralizes IL-11. The effect of delivered antibody mRNA using ionizable LNPs (mIL11-scFv@AA3G) lasted 18 times longer than that of IL11-scFv antibody alone (9 days vs. 12 h after injection) even at a lower dosage, and it alleviated hepatocyte metabolic dysfunction and HSCs activation by preventing c-Jun N-terminal kinases (JNK) and extracellular signal-regulated kinase (ERK) signaling induced by IL-11. Authors[574] also employed polymeric NPs modified with aminoethyl anisamide (chemically synthesized target ligands to activated HSCs) loaded with siRNA against *IL11 or IL11ra1* (siIL11@NP-AEAA or siIL11ra1@NP-AEAA, respectively) to fibrotic liver. Inhibition of IL11 signaling by siIL11ra1 @NP-AEAA demonstrated a superior therapeutic effect compared to siIL11@NP-AEAA in terms of reducing liver steatosis and fibrosis, as well as restoring liver function. He et al.[369] used MTC-conjugated NPs containing TNF-α-siRNA and alleviated fibrosis through the alternatively activated macrophages, protecting the liver from inflammation-induced damage (Fig. 13). Notably, this NP formulation demonstrated a higher TNF-a knockdown efficiency in macrophages in vitro compared to Lipofectamine2000 at an equivalent siRNA dose and also provided efficient oral delivery.

[575] used cationic solid lipid nanoparticles (CSLNs), reconstituted from natural LDLs and loaded with siRNAs against connective tissue growth factor (*CTGF*), which significantly reduced the collagen content and pro-fibrogenic factors such as TNF-α, TGF-β, IL-6, and CTGF, dramatically improving the pathophysiological symptoms in liver fibrosis model rats. Jiang et al.[576] designed lentiviral vector for delivery of artificial miR-30a and shRNAs for HSC-specific co-silencing of platelet-derived growth factor receptor beta (*PDGFR*-β) and *TGF-β2*, which remarkably inhibited profibrotic markers such as α-SMA and COL1α1, different types of matrix metalloproteinases (MMPs) and tissue inhibitors of metalloproteinases (TIMPs), and attenuated hepatic fibrosis in mice.

The second strategy is based on the regulation and resorption of excess ECM and collagen deposits in liver tissue. Several microRNAs, such as the let-7, miR-133a, miR-21, miR-378, miR-222, miR-30a, miR-24, miR-29a, miR-29b, and miR-122 family, were reported to be involved in the pathogenesis of liver fibrosis, because they modulate the expression of fibrogenesis-related genes, Snail and the Fas ligand, as well as a wide range of other proteins involved in the progression of fibrosis [577].

Among these microRNAs, miR-29b and miR-122 are the most promising therapeutic targets for liver fibrosis. The miR-29b and miR-122 inhibit collagen production by HSCs via targeting different specific signaling pathways of liver fibrosis. Moreover, it is anticipated that a combination of these two microRNAs may have an even larger synergistic antifibrotic effect [578].

**Fig. 14:**
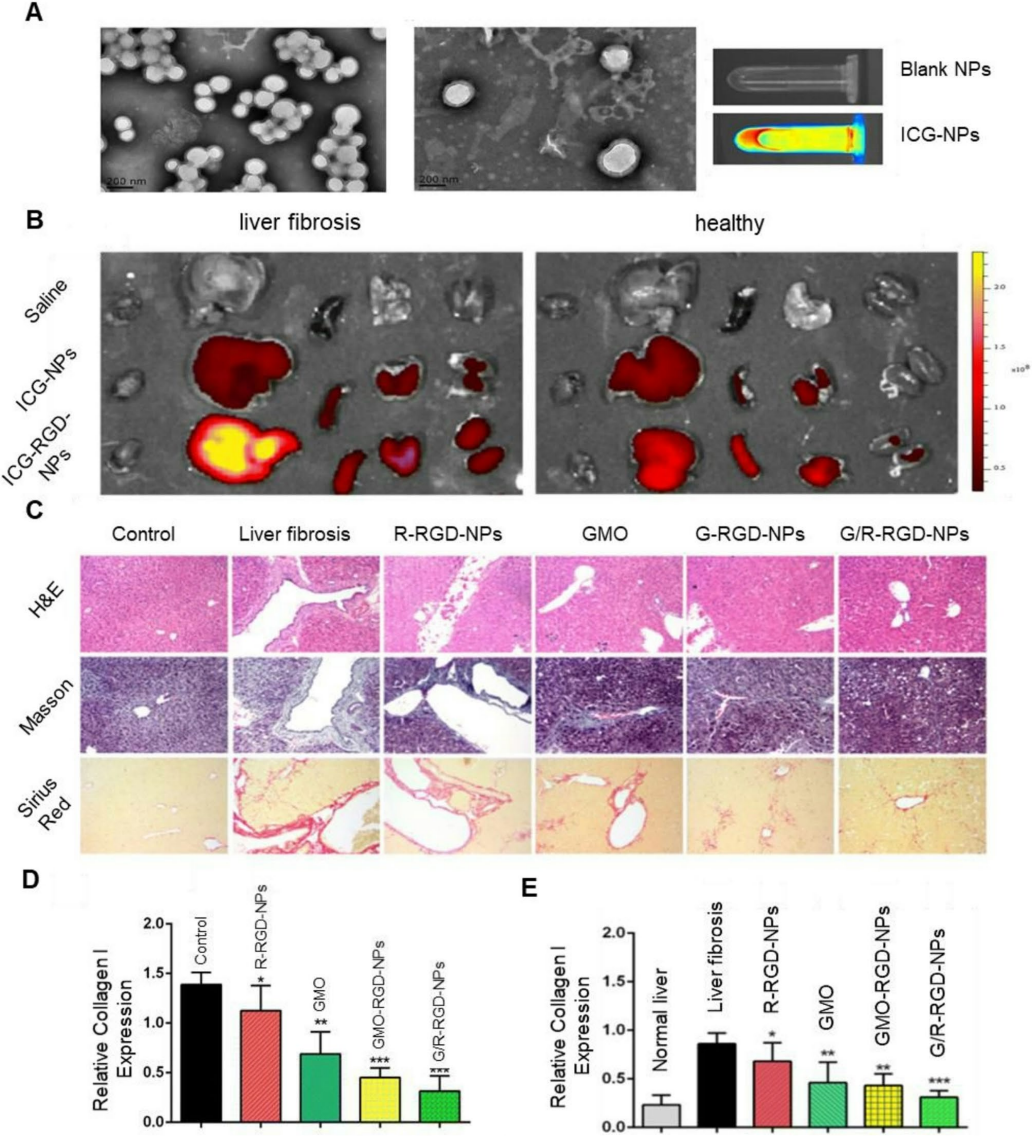
Enhanced accumulation and antifibrotic efficacy of RGD-conjugated nanoparticles in liver fibrosis models: **A** TEM images of G/R-NPs and G/R-RGD-NPs. Fluorescent images of blank NPs and ICG-RGD-NPs obtained using a Maestro imaging system. **B** Biodistribution of ICG-NPs and ICG-RGD-NPs in mice with Liver fibrosis and Healthy mice after 24 h. **C** H&E, Masson and Sirius Red staining of livers collected from healthy and liver fibrotic mice. **D **and ** E** Quantitative analysis of type I collagen after treatment in activated HSCs and fibrotic livers respectively (*p < 0.05, **p < 0.01, and ***p < 0.001 vs. control). Reproduced under terms of the CC-BY license [583]. 2020, De Ji et al., published by Springer Nature.

In detail, the miR-29b inhibits fibrogenesis by downregulating crucial regulators such as ECM proteins, indirectly blocking TGF-β1/Smad3 or hedgehog signaling pathway, and upregulating PTEN signaling cascade[579, 580]. miR-29 is currently the most widely studied as a promising antifibrotic agent. HSC-targeted combination therapy using miR-29b and miR-122 demonstrated significant antifibrotic efficacy, improving liver function and reversing liver fibrosis [578]. Moreover, Matsumoto et al. [581] reported that miR-29a can promote the recovery of the liver fibrosis induced by CCl4 or thioacetamide by regulating the expression of fibrosis-associated genes (*COL1A1* and *PDGF-C* mRNA). Kumar et al. [582] showed that co-delivery of small molecule hedgehog inhibitor and miR-29b targeting several profibrotic genes, including *COL1A1*, *PDGFR*-*β*, and *α*-*SMA*, provided effective therapy of bile duct ligation-induced liver fibrosis in mice. Ji et al. [583] showed targeted miR-29 and germacrone co-delivery to HSCs using PEGylated PLGA NPs modified with RGD peptides (RGD peptide: arginine (Arg), glycine (Gly), and aspartic acid (Asp)). Authors confirmed its cytotoxicity to activated HSCs, significantly inhibiting the production of type I collagen (Fig. 14).

A large number of less studied microRNAs were described to be playing different roles during the progression of liver fibrosis. For example, overexpression of the miR-199 and miR-200 families was found to be positively correlated with the TGF-β/SMAD signaling pathway during liver fibrogenesis in both mouse and human fibrosis models [546]. Brandon-Warner et al.[584] used AAV vector to deliver miR-19b under control of a collagen promoter targeted to HSCs, reduced fibrosis and Hepatic injury in rat model. After 2 weeks, authors demonstrated a ∼50% reduction in collagen deposition (compared to control animals) and significantly reduced hepatic injury with improvements in the total and direct serum bilirubin levels. miR-125b is another upregulated microRNA that promotes HSC activation via the RhoA (Ras Homolog Family Member A) signaling pathway, and inhibition of miR-125b attenuated liver fibrosis in vivo. Other microRNAs, including miR-214 [585–587], miR-181 [588–590], miR-34a[591–594], miR-652 [595–597], and miR-571[596, 598, 599] have also demonstrated significant potential as biomarkers and therapeutic targets for liver fibrosis and cancers.

Some drugs against fibrosis are already in the clinical trials stage, e.g., vitamin A-coupled LNPs loaded with siRNA BMS-986,263 targeting Heat shock protein 47 (HSP47, collagen-specific chaperone expressed in HSCs) (NCT02227459). The disruption of collagen synthesis via depletion of HSP47 protein decreased liver fibrosis in HCV-infected patients with advanced liver fibrosis (Metavir F3-4) [600]. According to the latest data, a phase 2 clinical trial is currently underway to evaluate the safety and efficacy of BMS-986,263 in patients with NASH and compensated cirrhosis of the liver (NCT04267393). Another fibroblast-targeting siRNA delivery platform [601] based on anisamide ligand-tethered lipidoids (AA-lipidoids) enables ~ 65% silencing of HSP47, notably reducing collagen deposition in fibrotic Liver. This promising platform has achieved 2-fold more efficient silencing than the FDA-approved MC3 ionizable lipid.

MMP-2 and MMP-14 are highly expressed in activated HSCs, promoting their migration and proliferation. MMP-2-siRNA delivered via vitamin A-linked liposome significantly reduced the expression of type I collagen and α-SMA in HSCs [602]. Halimani et al. [545] employed LNPs to deliver hyaluronan synthase 2 (*HAS2*) targeting siRNA, which significantly reduced the deposition of ECM in vivo. Qiao et al. [603] demonstrated that modified Chol-PEG-VA NPs with dual siRNA targeting *COL1Α1* and *TIMP-1* decreased liver fibrosis and markedly reduced the thickening of collagen bundles. The authors demonstrated high targeting efficiency for KCs under in vivo conditions, as well as the high efficacy of combined siRNA, which reduced collagen accumulation to the level observed in healthy mice without significant cytotoxicity. Kaps et al. [604] also developed cationic nanohydrogel particles to deliver COL1A1-siRNA for the treatment of CCl4-induced liver fibrosis, suppressing 50% of *COL1A1* mRNA in the fibrotic liver of mice.

Jain et al. [605] developed a PCBP2-siRNA hybrid nanocomplex (using siRNA/peptide nucleic acid (PNA) hybrid instead of chemically conjugated siRNA) and efficiently reversed CCl4-induced liver fibrosis in a rat model by preventing poly(RC) binding protein 2 (PCBP2)-mediated stabilization of *COL1A1* mRNA.

In addition to approaches that rely on suppression of pro-fibrotic signaling, ECM deposition and maturation, delivery of transcriptional factors (such as HNF4A, FOXA2, HGF, etc.) and anti-fibrotic hormones such as relaxin (RLN) also holds promise ([606]). Antifibrotic gene expression was also induced via delivery of LNPs loaded with *HNF4A* mRNA in vivo, reliably inhibiting fibrogenesis in 4 independent models of liver fibrosis in mice [607], [608] reported that *FOXA2* overexpression (via lentivirus or AAV8-mediated hepatocyte-specific delivery) alleviated the hepatic fibrosis. *FOXA2* overexpression in hepatocytes inhibited endoplasmic reticulum stress and hepatocyte apoptosis in CCL_4_-treated mice by activating the expression of a large number of metabolic genes. The HGF, traditionally considered a participant in liver regeneration, has also shown its effectiveness in anti-fibrotic therapy by inhibiting TGF-β signaling and HSC activation [609]. Lainscek et al. [610] showed that delivery of dCas9-VPR/sgRNA in a mouse model could counteract the liver damage by upregulation of the endogenous HGF. Luo et al. [611] used RBP4-modified exosomes for delivery of CRISPR/dCas9 system which induced the conversion from HSCs to hepatocyte-like cells via targeted activation of *HNF4α*/*HGF*/*FOXA2* genes, and demonstrated significantly attenuated CCl_4_-induced hepatic fibrosis. Relaxin is an antifibrotic peptide hormone that reverses the activation of HSCs and alleviates liver fibrosis [612]. Recombinant RLNs are currently undergoing clinical trials for the treatment of cardiac fibrosis [613]. Hu et al. [614]. used LNPs loaded with plasmid-encoded relaxin and mimics miR-30a-5p, which also demonstrated antifibrotic activity[615], and achieved a synergistic antifibrotic effect in a mouse model of liver fibrosis, successfully affecting and treating activated HSCs. The modified RLN-PLGF 1 chimeric protein improved the accumulation of RLN protein in the fibrous liver and increased antifibrotic activity in three NASH models. Shan et al.[616] developed a unique approach called «Fibrosis overexpression and retention» (FORT) based on a two-component system that included (1) retinoic acid LNPs that distribute retinoids on the surface of particles, facilitating RBP-4-mediated endocytosis and endosome release into HSC, and (2) modified mRNAs that encode the mRLN-CBD Fusion protein with the collagen-binding domain from placental growth factor PLGF 1, allowing attachment of expressed protein to fibrous ECM. Notably, the combination of mRLN-PLGF 1 and mIL-10-PLGF 1 LNP significantly improved liver fibrosis and inflammation. After combination therapy, the distribution of collagen in the liver was similar to that of the control group, and combination therapy almost eliminated the accumulation of lipid droplets.

#### Discussion: current limitations and research directions of fibrosis treatment

Fibrosis is often considered not as an independent disease, but as a manifestation of chronic liver pathologies, in particular NASH or viral hepatitis. Therefore, alleviation of the underlying pathology can also leads to resolution of fibrosis. But, complexity of the pathogenesis of fibrosis leads to difficulties in developing therapy, which should effect not only fibrous tissue, but also normalize lipid metabolism and reduce cell death [617–619]. Thus, it is promising to target several factors - HSC activation, the transmission of pro-fibrotic signals, and modulation of the cellular microenvironment, which improve the effectiveness of therapy. In addition to direct modulation of the activity of HSCs, the impact on other cells that actively interact with them is promising. For instance, preventing LSEC capillarization, which promotes HSC activation and impairs microcirculation, represents one such approach [620]. Liu et al. [621] developed spherical nucleic acid (SNA) NP carrying miR-325-3p, which specifically enters fibrotic LSECs via the scavenger receptor A (SCARA). The authors demonstrated the effectiveness of SNA - miR-325-3p in mouse models of liver fibrosis, showing restoration of LSEC fenestrations, reversed capillarization, and significantly reduced fibrosis without adverse effects. Preventing LSEC capillarization may also improve delivery of drugs and nanocarriers in advanced fibrosis [622], given that fibrosis markedly impaires the efficacy of gene therapy.

### Future prospects

Each of the considered liver pathologies involves many processes, including metabolic, immunological, and molecular genetic changes, constituting a complex pathophysiological pattern. Identification of key issues underlying these processes and treating them is a major area of ​​modern research. Methods of targeted therapy based on gene therapeutics, such as gene silencing or editing, allow selective treatment of these key nodes, minimizing the impact on other vital processes. Therapeutic strategies based on a combination of genomic medicine approaches or combined with classical treatment approaches have shown a powerful synergistic effect, which opens up new horizons in the treatment of liver pathologies. The enormous progress achieved in genomic medicine and the constantly growing number of approved therapeutic approaches and new fundamental discoveries deepen the understanding of liver diseases and enable the emergence of effective therapeutic tools for the liver pathologies previously considered difficult to be completely cured.

## Data Availability

No datasets were generated or analysed during the current study.

## Abbreviations

HSD17B13: 17β-hydroxysteroid dehydrogenase
FAE: A follicle-associated epithelium
HADHB: Acetyl-CoA acyltransferase
ACC: Acetyl-CoA carboxylase
ATF6: Activating transcription factor 6
ABEs: Adenine BEs
AAV: Adeno-associated virus
AdV: Adenovirus
a-SMA: Alpha smooth muscle actin
AMOs: Anti-miRNA oligonucleotides
ASGS: Antisense gene silencing
ASO: Antisense oligonucleotide
AGAP2: Arf GAP with GTP-binding protein-like domain, Ankyrin repeat and PH domain 2
Ago 1 to 4: Argonaute proteins
ABCXZ: TP-binding cassette transporters Subfamily X Member Z
BEs: Base editors
CROT: Carnitine O-Octanoyltransferase
CSLNs: Cationic solid lipid nanoparticles
CIDEC: Cell death inducing DFFA like effector C
CYP7A1: Cholesterol 7 alpha-hydroxylase
circRNA: Circular RNA
JNKs: C-Jun N-terminal kinases
SRBI: Class B type I scavenger receptor
CLDN: Claudin-1
CRISPR: Clustered regularly interspaced short palindromic repeats
CTGF: Connective tissue growth factor
CpAM: Core protein allosteric modulators
cccDNA: Covalently closed circular DNA
Cas: CRISPR-associated protein
DOCK11: Cytokinesis 11 dedicator
CBEs: Cytosine BEs
DAMPs: Damage-associated molecular patterns
dCas9: dead Cas9
DGAT2: Diacylglycerol acyltransferase 2
DsiRNAs: Dicer-substrate siRNAs
DSB: Double-strand DNA break
eTCR: Endogenous T cell receptor
ER: Endoplasmic reticulum
EGF: Epidermal growth factor
ECM: Extracellular matrix
ERK: Extracellular signal-regulated kinase
Fsp27: Fat-specific protein 27
FASN: Fatty acid synthase
FGF21: Fibroblast growth factor 21
FOXA3: Forkhead box A3
FoxM1: Forkhead box M1
GWAS: Genome-wide association studies
gRNA: Guide RNA
HSP47: Heat shock protein 47
HB-EGF: Heparin-binding EGF-like growth factor
HSCs: Hepatic stellate cells
HBsAg: Hepatitis B surface antigen
HBV: Hepatitis B virus
HCV: Hepatitis C virus
HCC: Hepatocellular carcinoma
HGF: Hepatocyte growth factor
HNF4a: Hepatocyte nuclear factor 4 alpha
HMGB1: High-mobility group box 1
HDR: Homology-directed repair
HITI: Homology-independent targeted integration
HAS2: Hyaluronan synthase 2
HIFs: Hypoxia-inducible factors
ILICI: Immune-mediated liver injury from immune checkpoint inhibitors
IRAK1: Interleukin 1 receptor associated kinase 1
IRES: Internal ribosomal entry site
KCs: Kupffer cells
LV: Lentivirus
LNPs: Lipid nanoparticles
LSECs: Liver sinusoidal endothelial cells
LNA: Locked nucleic acid
LDLR: Low density lipoprotein receptor
MRI: Magnetic resonance imaging
mTOR: Mammalian target of rapamycin
MTC: Mannose-modified trimethyl chitosan-cysteine
MMPs: Matrix metalloproteinases
MSNs: Mesoporous silica nanoparticles
MCJ: Methylation-Controlled J protein
MTARC1: Mitochondrial amidoxime reducing component 1
MT-CO2: Mitochondrially encoded cytochrome C oxidase II
MCP-1: Monocyte chemotactic protein 1
GalNAc: N-Acetylgalactosamine
NDUFS2: NADH:Ubiquinone Oxidoreductase Core Subunit S2
NP: Nanoparticle
NK: Natural killer
NAbs: Neutralizing antibodies
nCas9: Nickase Cas9
NLRP3: NLR family pyrin domain containing 3
NAFLD: Non-alcoholic fatty liver disease
NASH: Non-alcoholic steatohepatitis
NHEJ: Non-homologous end joining repair
NPCs: Non-parenchymal cells
NAPs: Nucleic acid polymers
OCLN: Occludin
PNPLA3: Patatin-like phospholipase domain containing 3
PLINs: Perilipins
PB: PiggyBac
PLGF 1: Placental growth factor 1
PDGFR-β: Platelet-derived growth factor receptor beta
PCBP2: Poly(RC) binding protein 2
PTGS: Post-transcriptional gene silencing
pgRNA: Pregenomic RNA
PD-L1: Programmed cell death 1 ligand 1
PD1: Programmed death factor-1
PCNA: Proliferating cell nuclear antigen
PCNAP1: Proliferating cell nuclear antigen pseudogene 1
PRK2: Protein kinase C-related kinase 2
rTCR: Recombinant T cell receptor
RLN: Relaxin
RES: Reticuloendothelial system
RhoA: Ras Homolog Family Member A
RNP: Ribonucleoprotein
RISC: RNA induced silencing complex
RdRp: RNA-dependent RNA polymerase
RITA: RNA-induced transcriptional activation
RUNX1: Runt-related transcription factor 1
S/MARs: Scaffold matrix attachment regions
saRNA: Self-amplifying sequences
Stk25: Serine/threonine protein kinase 25
PLK1: Polo Like Kinase 1
shRNAs: Short hairpin RNAs
SLB: Silibinin
scFv: Single-chain variable fragment
SB: Sleeping Beauty
saRNAs: Small activating RNAs
NTCP: Sodium/bile acid cotransporter
SCAP: SREBP cleavage activating protein
SCD1: Stearoyl-Coenzyme A desaturase 1
SREBPs: Sterol regulatory element-binding proteins
SVP: Subviral particles
TFV: Tenofovir
Smad: Small mother against decapentaplegic
TIMPs: Tissue inhibitors of metalloproteinases
TLRs: Toll-like receptors
TALEN: Transcription activator-like effector nuclease
TGF-β: Transforming growth factor beta
TM6SF2: Transmembrane 6 superfamily member 2
TEM: Transmission electron microscopy
TG: Triglycerides
TNF-α: Tumor necrosis factor
T2DM: Type 2 diabetes mellitus
VLDL: Very low-density lipoproteins
XBP1: X-Box binding protein 1
ZFN: Zinc finger nuclease
α-Gal: α-Galactosidase
